# Evolution of structural rearrangements in prostate cancer intracranial metastases

**DOI:** 10.1038/s41698-023-00435-3

**Published:** 2023-09-13

**Authors:** Francesca Khani, William F. Hooper, Xiaofei Wang, Timothy R. Chu, Minita Shah, Lara Winterkorn, Michael Sigouros, Vincenza Conteduca, David Pisapia, Sara Wobker, Sydney Walker, Julie N. Graff, Brian Robinson, Juan Miguel Mosquera, Andrea Sboner, Olivier Elemento, Nicolas Robine, Himisha Beltran

**Affiliations:** 1 Department of Pathology and Laboratory Medicine, Weill Cornell Medicine, New York, NY USA; 2 Englander Institute for Precision Medicine, Weill Cornell Medicine, New York, NY USA; 3 New York Genome Center, New York, NY USA; 4 Department of Medical and Surgical Sciences, Unit of Medical Oncology and Biomolecular Therapy, University of Foggia, Policlinico Riuniti, Foggia, Italy; 5 Department of Pathology and Laboratory Medicine, UNC Chapel Hill, Chapel Hill, NC USA; 6 Department of Medical Oncology, Oregon Health Sciences University, Portland, OR USA; 7 The HRH Prince Alwaleed Bin Talal Bin Abdulaziz Alsaud Institute for Computational Biomedicine, Weill Cornell Medicine, New York, NY USA; 8 Meyer Cancer Center, Weill Cornell Medicine, New York, NY USA; 9 Department of Physiology and Biophysics, Weill Cornell Medicine, New York, NY USA; 10 Department of Medical Oncology, Dana-Farber Cancer Institute, Boston, MA USA

**Keywords:** Prostate cancer, Cancer genomics

## Abstract

Intracranial metastases in prostate cancer are uncommon but clinically aggressive. A detailed molecular characterization of prostate cancer intracranial metastases would improve our understanding of their pathogenesis and the search for new treatment strategies. We evaluated the clinical and molecular characteristics of 36 patients with metastatic prostate cancer to either the dura or brain parenchyma. We performed whole genome sequencing (WGS) of 10 intracranial prostate cancer metastases, as well as WGS of primary prostate tumors from men who later developed metastatic disease (*n* = 6) and nonbrain prostate cancer metastases (*n* = 36). This first study focused on WGS of prostate intracranial metastases led to several new insights. First, there was a higher diversity of complex structural alterations in prostate cancer intracranial metastases compared to primary tumor tissues. Chromothripsis and chromoplexy events seemed to dominate, yet there were few enrichments of specific categories of structural variants compared with non-brain metastases. Second, aberrations involving the *AR* gene, including *AR* enhancer gain were observed in 7/10 (70%) of intracranial metastases, as well as recurrent loss of function aberrations involving *TP53* in *8/10 (80%), RB1* in 2/10 (20%), *BRCA2* in 2/10 (20%), and activation of the PI3K/AKT/PTEN pathway in 8/10 (80%). These alterations were frequently present in tumor tissues from other sites of disease obtained concurrently or sequentially from the same individuals. Third, clonality analysis points to genomic factors and evolutionary bottlenecks that contribute to metastatic spread in patients with prostate cancer. These results describe the aggressive molecular features underlying intracranial metastasis that may inform future diagnostic and treatment approaches.

## Introduction

Systemic therapy for metastatic prostate cancer has improved significantly over the last decade, leading to improvements in overall survival^1^. Likely due to more effective disease control and patients living longer, patterns of metastases have also evolved. Prostate cancer typically spreads to lymph nodes and bone. In later stages, visceral metastasis to liver, lungs, or bone marrow may be observed. Intracranial brain metastases, once thought to be rare in prostate cancer, are increasingly described, which may be due to better systemic disease control with drugs that do not cross the blood-brain barrier^2^. The absolute incidence and associated clinical characteristics of intracranial metastases in prostate cancer in the current era is not well established, but it is a major cause of morbidity and mortality for affected patients. In other solid tumors, brain metastases have been associated with distinct and potentially actionable genomic alterations not observed in primary tumor tissues^3–6^. Understanding patterns of tumor evolution can provide insights into clinical strategies for detecting, preventing, or treating brain metastases^7^.

Prostate cancer is characterized by a relatively low mutational burden and a predominance of copy number alterations, complex rearrangements, and structural alterations that are not often appreciated through exome sequencing^8^. Here we performed whole genome sequencing (WGS) of a cohort of primary and metastatic prostate cancers, including intracranial prostate cancer metastases, to identify the spectrum of genomic alterations present in prostate cancer intracranial metastases and their concordance with other sites of disease.

## Results

### Clinical Features

We identified 36 patients with prostate cancer intracranial metastases (33 were from 2010 to 2018; 4 cases before 2010 (2003–2009)). These were further classified as parenchymal brain (*n* = 22, 61.1%), dural-based (*n* = 13, 36.1%) or both (*n* = 1, 2.8%). The median time from prostate cancer diagnosis to intracranial metastasis was 56.6 months. Prostate cancer grade at diagnosis was Grade Group 4 (GG4) or higher in 15 cases (41%), lower than GG4 in 11 cases (31%), with data on grade group unavailable from the remaining 10 cases (28%). The median number of lines of systemic therapy given for metastatic disease before intracranial metastasis was three (range 0–8). One patient presented with multiple de novo parenchymal brain metastases without any prior therapy for prostate cancer or other sites of metastasis. Median serum prostate specific antigen (PSA) level at the time of intracranial metastases was 50 ng/mL (range 4.32–4308 ng/mL). Additional sites of metastases at the time of intracranial metastasis included bone (88.9%), lymph node (33.3%), lung (30.5%), liver (25%), and other sites (16.7%). Median overall survival after the development of intracranial metastasis was 11.2 months. Clinical characteristics are summarized in Table 1.

**Table 1:** Clinical features of prostate cancer patients with brain metastases.

Characteristics of overall patients n (%)	36 (100%)
Age at the time of brain metastases, years Median (range)	66 (50–86)
Gleason score at diagnosis, *n* (%)	
<8	11 (42.3)
≥8	15 (57.7)
Missing	10
Histology, *n* (%)	
Adenocarcinoma	28 (77.8)
NEPC	8 (22.2)
Prostatectomy, *n* (%)	
No	24 (66.7)
Yes	12 (33.3)
Radical radiotherapy, *n* (%)	
No	14 (38.9)
Yes	22 (61.1)
Site of brain metastasis, *n* (%)	
Parenchymal	22 (61.1)
Dural	13 (36.1)
Both	1 (2.8)
Type of brain metastasis, *n* (%)	
Solitary	22 (61.1)
Multiple	14 (38.9)
Other sites of metastasis at the time of brain met diagnosis, *n* (%)	32 (88.9)
Bone	12 (33.3)
Nodal	11 (30.5)
Lung	9 (25.0)
Liver	6 (16.7)
Other	
Number of prior systemic therapies before brain metastasis diagnosis	
Median (range)	3 (0–8)
Prior treatment with abiraterone or enzalutamide, *n* (%)	13 (36.1)
PSA at the time of brain metastasis, ng/mL Median (range)	50 (4.32–4308)
Serum chromogranin at the time of brain metastasis, *n* (%)	
< 95 ng/mL	8 (44.4)
> 95 ng/mL	10 (55.6)
Serum NSE at the time of brain metastasis, *n* (%)	
< 8.9 ug/L	8 (44.4)
> 8.9 ug/L	10 (55.6)
Time from prostate cancer diagnosis to brain metastasis, months	56.6 (0.6–253)
Median (range)	
Overall survival from time of metastasis, months Median (range)	11.2 (0.6–59.9)
Parenchymal	16.8 (1.9–42.5)
Dural	10.6 (0.6–59.9)

^#^ Upper normal limit.

*CGA* chromogranin A, *CRPC* castration-resistant prostate cancer, *NEPC* neuroendocrine prostate cancer, *NSE* neuron specific enolase, *PSA* prostate specific antigen.

### Histological and immunohistochemical features

**Fig. 1:**
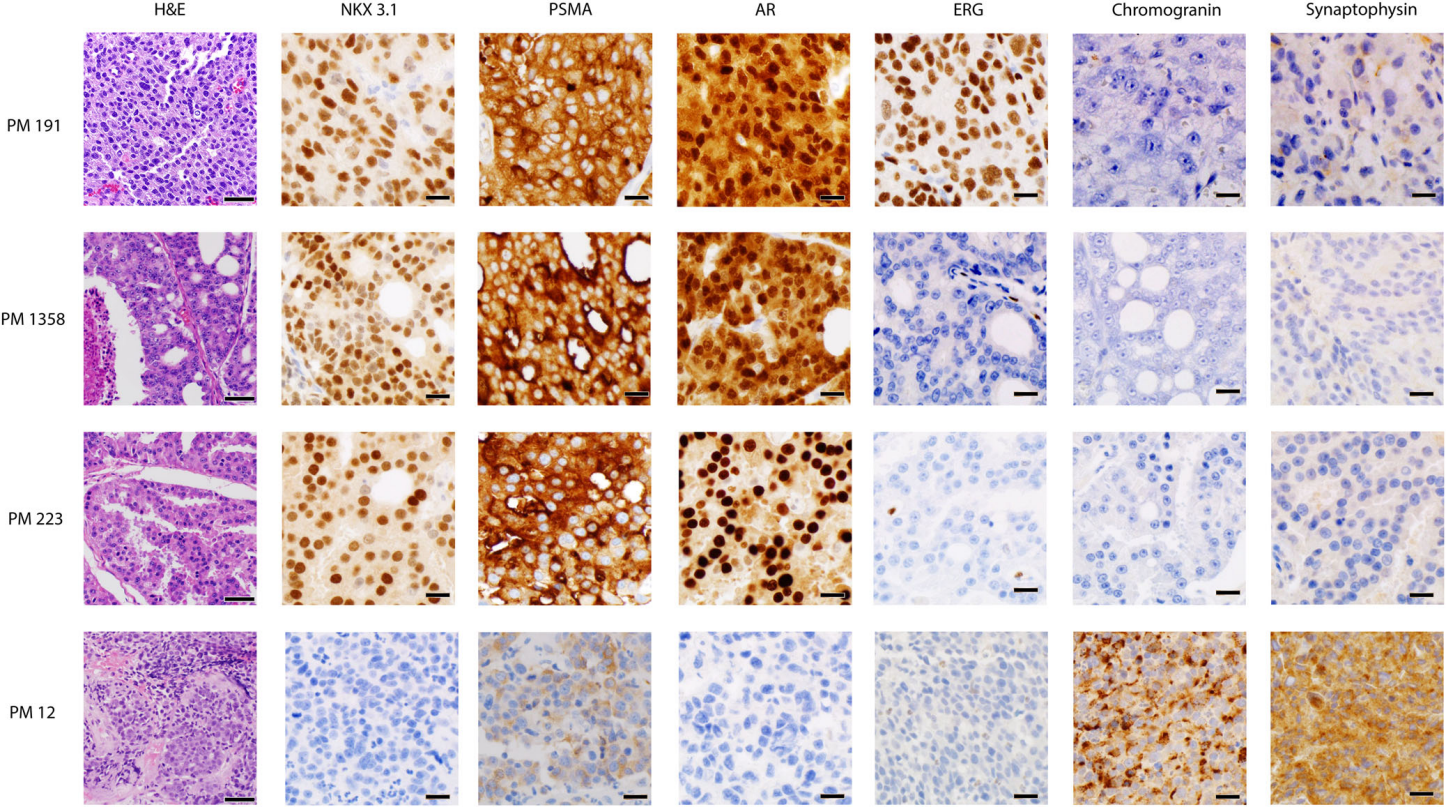
Histomorphology and immunohistochemical expression of intracranial metastatic prostate cancer samples. Representative H&E-stained sections (shown at 200x magnification, scale bar = 50 uM) and immunohistochemical stains (shown at 400x magnification, scale bar = 20 uM) of four representative intracranial metastatic samples. WCM191 exhibited a solid sheet of prostatic adenocarcinoma cells with nuclear pleomorphism and was strongly positive for NKX3.1, PSMA, AR (both nuclear and cytoplasmic staining), showed ERG overexpression, and was negative for neuroendocrine markers, chromogranin and synaptophysin. WCM1358 was a prostatic adenocarcinoma with large cribriform architecture, necrosis, and prominent nucleoli, with a similar immunohistochemical staining pattern to WCM191 except for the lack of ERG expression. WCM223 was also a prostatic adenocarcinoma predominantly with micropapillary and focally cribriform architecture, with similar staining pattern to WCM1358 but showing predominantly nuclear AR staining. WCM12 was a treatment-related NEPC, showing a solid sheet of tumor cells with a high nuclear to cytoplasmic ratio, abundant mitotic activity, and a lack of prominent nucleoli. This tumor was positive for chromogranin and synaptophysin, and was negative for NKX3.1, focally positive for PSMA, and negative for AR and ERG.

For twenty patients, metastatic intracranial tumor resection was performed clinically for a solitary or dominant brain lesion or obtained at the time of rapid autopsy^9^. In 19/20 cases, the intracranial metastases were classified as high-grade acinar adenocarcinoma. Morphologically, these cases exhibited varied patterns including solid sheets of tumor cells, dense and loose cribriform or micropapillary architecture, and/or poorly-formed glands, with varying degrees of nuclear pleomorphism, mitotic activity, and necrosis (Fig. 1). One patient who had two serial metastatic brain samples, with the second obtained from a second surgery for relapsed disease, had treatment-emergent neuroendocrine prostatic carcinoma (NEPC)^10,11^ based on tumor morphology with similar morphology in both tumor resections (Fig. 1). The histologic and immunohistochemical features of representative intracranial metastases are demonstrated in Fig. 1.

For all intracranial metastases with high grade adenocarcinoma, immunohistochemical (IHC) staining for NKX3.1 and AR were strongly and diffusely positive. IHC staining for PSMA was positive (>50% of cells staining) in 6/12 of these cases and showed patchy, weak, or focal staining in the remaining six cases. Chromogranin and synaptophysin expression were negative in 11/12 of cases and focally positive in one case. ERG overexpression by IHC was observed in 3/12 cases. The one case of intraparenchymal brain metastases with treatment-emergent NEPC diffusely expressed the neuroendocrine markers chromogranin and synaptophysin, demonstrated weak positivity for PSMA, and was negative for NKX3.1, AR, and ERG protein expression^1^ (Fig. 1), in concordance with this diagnosis.

### Whole genome sequencing of prostate cancer primary tumors and metastases

We performed whole genome sequencing (WGS) of primary prostate tumors from men who later developed metastatic disease (*n* = 6) and castration resistant metastatic prostate tumors from various anatomic sites (*n* = 46), including intracranial metastases (*n* = 10). Patient characteristics are summarized in Supplementary Table 1. Matched germline DNA was also sequenced. Median WGS coverage for the cohort was 96X (tumor) and 48.5X (normal).

**Fig. 2:**
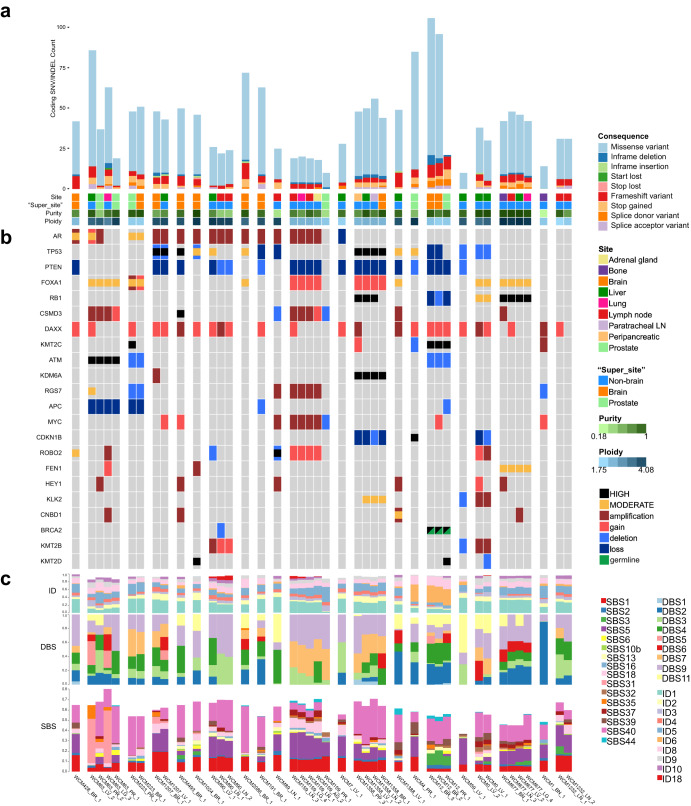
The landscape of small variants in metastatic prostate cancer. **a** Coding mutation burden, split by Ensembl coding consequence. Missense mutations are excluded from the plot. Samples are grouped by patient and sorted according to the below oncoprint. **b** Oncoprint of focal copy changes, as well as moderate and high-impact SNV/INDELS, sorted by variant prevalence across the cohort and color coded by variant category. Copy number variants larger than 3MB in length are excluded from the figure. **c** Proportions of COSMIC insertion/deletion (ID), doublet base substitution (DBS), and single base substitution (SBS) mutational signatures. SBS signatures identified as potential artifacts in COSMIC are excluded from the figure.

**Fig. 3:**
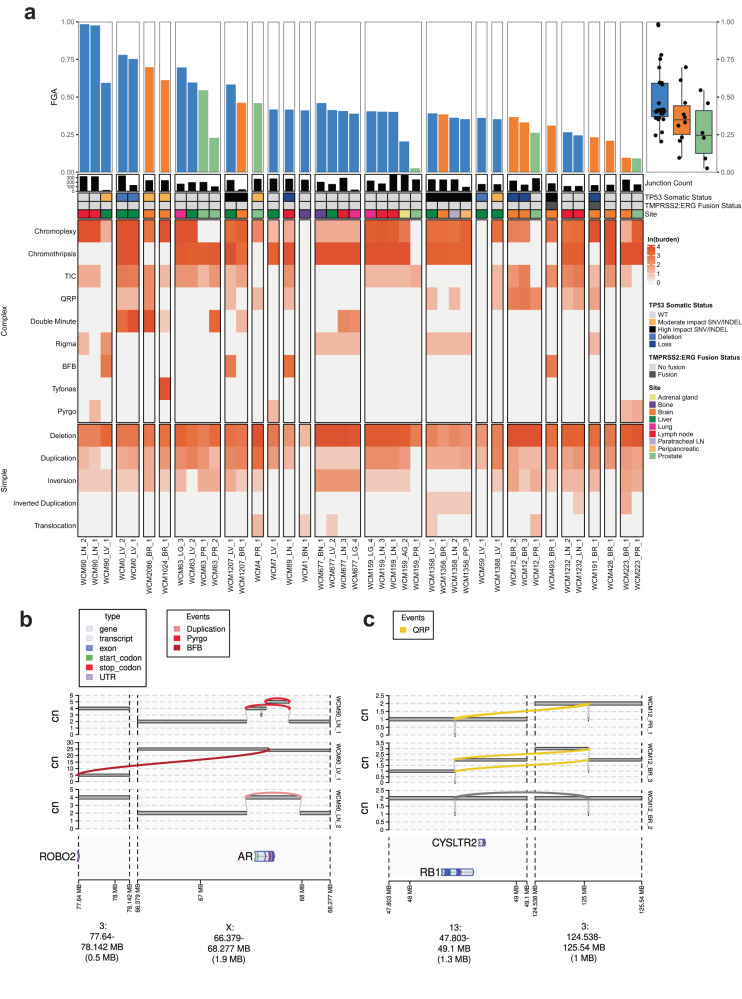
High diversity and burden of complex structural alterations. **a** Top: barplot representing the fraction of the genome altered (FGA), grouped by patient and sorted by median FGA, and including singletons. The right-hand panel shows a summary of FGA by tumor localization: prostate, brain, or nonbrain. Bottom: Heatmap of junction burden assigned to each JaBbA event type, grouped by simple and complex event types. Cells are colored by the natural logarithm of the number of junctions associated with that sample/event combination. **b** JaBbA genome graph showing convergent evolution at the AR locus across three samples from a single patient. The horizontal bars represent genomic regions, and their height represents their clonal copy number. Reference adjacencies are indicated by thin grey lines connecting segments, and non-reference adjacencies (e.g. structural variant breakpoints), are colored by the JaBbA event they are part of. A simple duplication, breakage-fusion-bridge, and pyrgo events are observed. The location of AR and is indicated at the bottom of the panel. **c** JaBbA genome graph showing the complex evolutionary pattern of a structural variant disrupting RB1.

Common recurrent aberrations in primary tumors and metastatic lesions are shown in Fig. 2. In primary tumors, recurrent aberrations included *FOXA1* mutation (3/6 samples, 2/5 patients), homozygous or heterozygous deletion of *APC* (4 samples, 3 patients), one heterozygous and one homozygous *PTEN* deletion (2 samples, 2 patients), *ATM* deletion (2 samples, 2 patients) or mutation (2 samples, 1 patient), and breakpoint disruption resulting in partial copy number gain of *CSMD3* (2 samples, 1 patient). Recurrent alterations in prostate cancer metastases included *FOXA1* mutation (11/36 samples, 6/20 patients), *TP53* mutation (11 samples, 7 patients), *RB1* mutation (9 samples, 3 patients), homozygous or heterozygous deletion of *PTEN* (21 samples, 11 patients), copy number gain of *MYC* (9 samples, 6 patients) and *FOXA1* (8 samples, 2 patients), breakpoint disruption resulting in heterozygous deletion of *TP53* (7 samples, 5 patients) and breakpoint disruption resulting in partial copy gain of *CSMD3* (8 samples, 4 patients). Overall, these recurrent genomic alterations were similar in frequency to prior exome-based metastatic castration resistant prostate cancer (CRPC) sequencing studies^12–14^. The *AR* gene was amplified via a variety of structural aberrations, including double minutes, breakage-fusion-bridge cycles, pyrgo, simple duplications, and other complex rearrangements not classified by the JaBbA algorithm (see Methods) (16 samples, 10 patients) (as exemplified in Fig. 3c) (Supplementary Fig. 1). We detected a median of 5507 (range 1350–15708) noncoding mutations across the cohort, with no significant difference in noncoding burden between prostate and metastatic tumors (Two-sided Wilcoxon rank-sum test, *p* = 0.96) (Supplementary Fig. 2). After accounting for mutations shared between samples from the same patient, 49 noncoding mutations were shared within metastatic tumors, with no mutations shared by more than two patients (Supplementary Table 2). No noncoding mutations were shared between prostate tumors. 16 noncoding mutations were common to both the prostate and metastatic groups. Overall, the vast majority (99.96%, 171508/171573) of noncoding mutations were unique to individual patients.

### Intracranial metastases compared with other metastatic lesions

There was no significant difference in mutation rate, proportion of mutational signatures, or frequency of mutation in individual genes between intracranial metastases and other metastatic sites of disease (Fig. 2a). Recurrent somatic alterations in intracranial metastases did not reach statistical significance for enrichment and included *TP53* mutation or deletion (8/10 samples, 7/9 patients with intracranial metastases versus 10/26 samples, 7/13 patients with other metastatic lesions), *AR* mutation (2 samples, 2 patients) and *AR* full or partial copy number gain (6 samples, 6 patients), *FOXA1* mutation or gain (3 samples in 3 patients), homozygous or heterozygous deletion of *PTEN* (7 samples in 6 patients), and *SKI* (4 samples, 3 patients). *AR* enhancer amplification (7 samples, 7 patients) frequently co-occurred with focal *AR* amplification (6/7 samples, 6/7 patients), but not *AR* mutation (2/7 samples, 2/7 patients). Loss of function aberrations involving one or more of the tumor suppressor genes, *TP53*, *PTEN*, or *RB1*, was present in 9/10 brain metastases and loss of two in 4/10, with two samples from the patient with treatment-emergent NEPC (WCM12) harboring loss of all three.

### Genome-wide features of tumor evolution

The distribution of mutational signatures (predominance of signatures SBS1 and 5 (“clock-like” signatures, associated with aging), as well as SBS40 (unknown etiology)) (Fig. 2c) in primary tumors and metastases was consistent with what has been reported in prostate cancer (TCGA). One patient with both a germline and a somatic *BRCA2* alteration displayed a mutation pattern corresponding to the signatures associated with homologous recombination deficiency (primarily SBS3 and ID6)^15^ (Fig. 2c). We note that 7 of the 10 brain metastatic samples presented a mutation or a copy number alteration (loss of function/deletion) in one of the 15 homologous recombination repair (HRR) genes included in the PROfound clinical trial^16,17^ (Supplementary Fig. 3).

We quantified the fraction of genome altered (FGA) as a proxy for chromosomal instability (CIN) and identified a systematic augmentation of FGA in metastases compared to their matched primary tumors, as well as an important per-patient variation (Fig. 3a). Our results do not indicate a higher chromosomal instability in brain metastasis compared to nonbrain metastatic samples.

We leveraged whole-genome data to look more closely at structural variants and somatic DNA rearrangement junctions in primary and metastatic tumors (Fig. 3b), including both simple (eg., deletions, translocations) and complex (e.g., chromothripsis, chromoplexy, complex rearrangements) events. Chromothripsis and chromoplexy seemed to dominate metastatic samples, though there was also enrichment of specific categories of structural variants including double-minutes events, templated insertion chains (TIC) and other complex structural variants. Taking all classes of structural variants into consideration, we noticed a larger diversity of structural variant classes in both intracranial and non-brain metastases compared with primary tumors.

One patient (WCM90) with 2 metastasis samples from lymph nodes and one from the liver show independent *AR* amplification. The two lymph nodes samples (LN_1 and LN_2) had a similar focal amplification (estimated Copy Number = 4), while the liver sample had a large amplification (CN ~ 40) (Fig. 3c). These events must have occurred independently and represent an example of convergent evolution.

In one patient (WCM12) we sequenced a primary prostate tumor sample (PR1; a high grade adenocarcinoma with focal neuroendocrine differentiation) and two intracranial metastases (treatment-related NEPC) samples obtained from the same location 3 months apart, with the second sample (named BR2) likely to correspond to a recurrence of the original metastatic sample (BR3). We identified a complete inactivation of *RB1* in the primary tumor, with one allele deleted and the second disrupted by a Quasi-Reciprocal Pair (akin to a reciprocal translocation with a short gap between both breakpoints) with a genomic region of chromosome 3. In the first brain metastatic sample, one side of the translocation (containing the 3’ end of *RB1*) gained a copy. In the second resurgent metastasis, the intact allele of the translocation partner was deleted and the other side of the translocation (with the 5’end of *RB1*) was amplified (Fig. 3d). We cannot identify the order of these events or determine if they had already occurred when the first metastasis was resected. *RB1* loss of function is infrequent in primary untreated prostate cancer and is associated with poor prognosis in patients with metastatic CRPC, in part due to its key role in lineage plasticity and NEPC progression^14,18,19^. These data support mechanisms that inactivate *RB1* that would not have been appreciated by exome or targeted sequencing.

### Genomic sequencing of other disease sites in patients with brain metastases

**Fig. 4:**
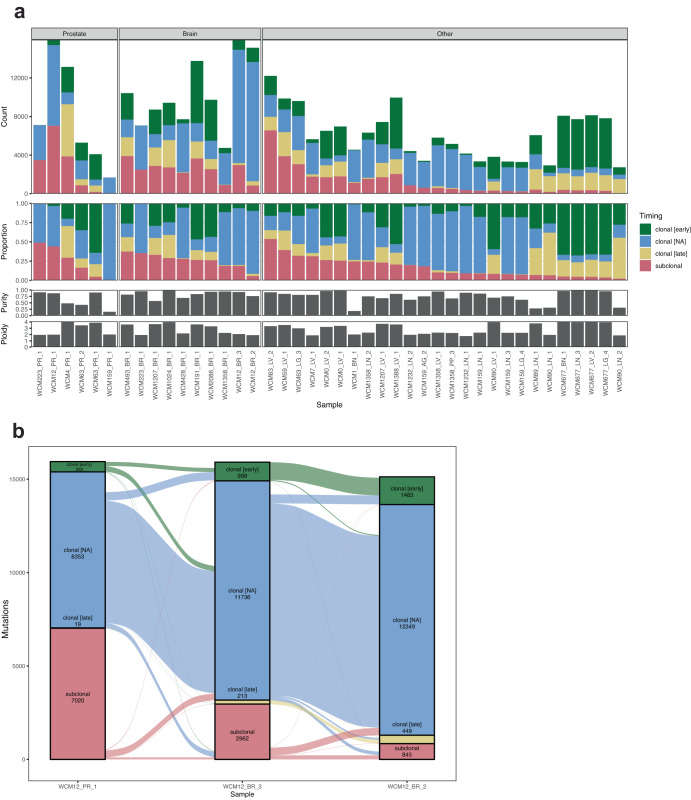
Clonal heterogeneity and evolution. **a** Barplots summarizing SNV/INDEL timing for each sample, grouped by localization. Raw counts are shown in the top track, with proportions shown directly below. The bottom two tracks indicate sample purity and ploidy. **b** The clonal evolution of variants shared between a prostate primary and brain metastasis sample from the same patient. The alluvial plot summarizes the timing for private and shared variants, where the magnitude of shared variants indicated by the width of the alluvia.

The concordance of alterations between intracranial metastases and other metastatic sites was evaluated in four individuals (WCM12, WCM223, WCM63, WCM159). The overall mutational and copy number profiles did not significantly differ across disease sites. These data imply the presence of multiple somatic alterations already present in metastatic CRPC tumors that are maintained in intracranial metastases. By applying an algorithm to estimate mutational timing based on copy-number and allelic fraction (MutationTimer, see Methods), we determined the clonal or subclonal status of all somatic SNV and indels. The proportion of subclonal mutations was not different between brain and non-brain metastasis (Supplementary Fig. 4) but was notably higher in the primary tumors of the two patients with brain metastasis (WCM12 and WCM223) than in the two patients with non-brain metastasis (WCM63 and WCM159, Fig. 4a). We decomposed the subclonal mutations into the known COSMIC signatures but were not able to detect subclonal signatures specific to intracranial metastases (Supplementary Fig. 5). Besides, the accumulation of clonal or subclonal mutations specific to the metastases partially reflect the time between the seeding and the sampling, which cannot be controlled for. We focused our attention on the shared variants between primary and metastases. In patient WCM12, 83% of mutations classified as “subclonal” in the primary tumor and conserved in intracranial metastases became “clonal”, suggesting that the seeding of this metastasis was monoclonal, and that the other subclonal mutations in the metastasis occurred after seeding. In the relapsed metastatic sample, a similar proportion of subclonal variants became clonal, suggesting a similar evolutionary bottleneck (Fig. 4b). In another patient (WCM223) with a primary tumor and an intracranial metastasis, half of the subclonal mutations observed in the primary and conserved in the metastasis changed status to “clonal”, while the other half remained subclonal. In these two patients, a substantial proportion of the subclonal mutations are found in the brain metastasis, indicating that the seeding occurred when the primary tumor was already heterogeneous and already contained subclonal mutations. In the two patients with primary and non-brain metastases (WCM159 and WCM63), very few mutations in the primary tumor were classified as “subclonal: therefore could not inform about monoclonal or polyclonal seeding of the metastasis (Supplementary Fig. 6).

### Gene expression of brain and non-brain metastases

**Fig. 5:**
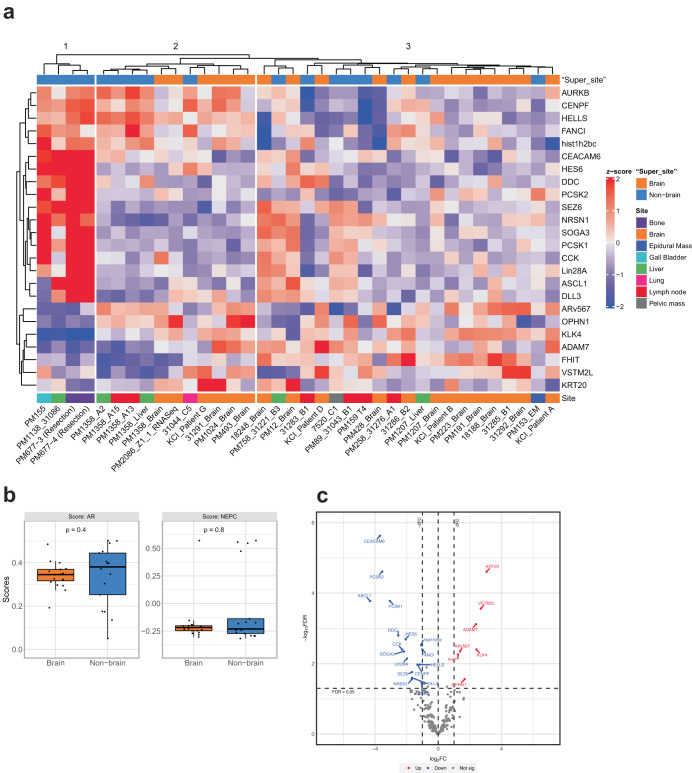
Gene expression of intracranial metastases. **a** Hierarchical clustering heatmap analysis of DEGs between Brain and Non-brain metastases revealed 3 clusters. Red in the heatmap denotes upregulation while blue denotes downregulation. Horizontal axis represents the metastatic samples and the vertical axis denotes the DEGs comparing Brain to Non-brain metastases. Dendrograms for samples and genes are shown in the heatmap. Values are measured by Euclidean distance with a complete linkage clustering algorithm. **b** The association between risk scores and clinicopathological information was evaluated and presented as a box plot. The horizontal line in each box represents the median for each group. The bottom and top of each box represent the first and third quartiles, respectively. The vertical lines extend to the values no farther than 1.5 times the interquartile range. **c** Volcano plot comparing intracranial metastases (*n* = 19) with other metastatic sites of disease (*n* = 17). Red dots: differentially expressed genes that were significantly upregulated in intracranial metastases (log_2_FC > 1, padj < 0.05). Blue dots: differentially expressed genes that were significantly downregulated in intracranial metastases (log_2_FC < (−1), padj < 0.05). Black dots: statistical tests were not significant.

Gene expression of prostate cancer intracranial metastases (*n* = 20) and their matched primary tumor (*n* = 7) or other patient-matched metastatic sites (*n* = 5) was evaluated by a custom-designed panel of 361 prostate cancer-related genes using the Nanostring platform (Supp. Table 2). To compare relative gene expression, these data were evaluated in the context of previously published data using the same platform of primary prostate cancer and CRPC (Supplementary Fig. 7). There was high concordance of mRNA expression by Nanostring with RNAseq and protein expression by IHC for AR, CHGA, SYP, ERG, NKX31, PSMA, and RB protein (Supplementary Fig. 8). Unsupervised clustering of brain and nonbrain samples based on the targeted prostate cancer panel revealed three distinct clusters with segregation of samples based on the patient rather than site of metastasis (Fig. 5a). We observed that AR expression and canonical AR signaling score were high in all intracranial metastases, and neuroendocrine marker expression, as well as NEPC signaling score were low, with exception of the one treatment-emergent NEPC brain metastasis (Fig. 5b). Comparison of intracranial metastases with other metastatic sites of disease revealed differentially expressed genes in brain metastases (Fig. 5c), including upregulation of the type 1 cytokeratin *KRT20* (CK20); *ADAM7*, a protease implicated in cancer progression; *AR-*regulated genes (KLK4, ARv567); and *OPHN1* (located at the same region as *AR* gene); and downregulation of the cell adhesion marker *CEACAM6*, and neuroendocrine associated genes *PSCK2* and *ASCL1*. Altogether, these results suggest that while brain metastases maintain the expression profile of the original tumor, they also may acquire a brain metastasis-associated gene expression program.

## Discussion

Intracranial metastases are considered rare in prostate cancer, though increasingly recognized likely as a result of patients living longer with more effective systemic disease control. Intracranial metastases in prostate cancer can be further classified anatomically as involving the brain parenchyma (arising through hematogenous spread with disruption of the blood-brain barrier) or as dural-based (from hematogenous spread, or through direct extension from adjacent involvement of the skull or epidural disease). Both types of intracranial metastases can result in neurologic symptoms and significant morbidity and mortality for patients. According to prior rapid autopsy studies, less than 10% of patients with late-stage prostate cancer harbored intracranial metastases and most of these were dural-based^20^. However, most of these studies were conducted before the introduction of several contemporary life prolonging agents for CRPC. Recent clinical reports have suggested a relative increase in brain metastases^2,17^, but the exact incidence and factors associated with the development of intracranial metastases have not been fully defined. In our current study, the presence of either parenchymal or dural intracranial metastases was associated with poor prognosis.

Little is known about the molecular features of intracranial metastases in prostate cancer, which may be due to their relative infrequency and their inaccessibility for tumor evaluation. Consistent with a recent report by Rodriguez-Calero et al.^17^, we identified frequent DNA repair aberrations in intracranial metastases. Based on their clinical aggressiveness, we had posited that intracranial metastases would represent tumors at the end of the spectrum and may demonstrate features of AR-independent disease. However, our results here point to continued AR signaling activation in the cases we analyzed, with frequent *AR* gene aberrations (>70%).

The overall similarity of intracranial metastases with other sites of metastases in individual patients suggests that while intracranial metastases have widely aberrant genomes, most alterations likely occurred prior to intracranial metastases and may have been facilitators of widespread dissemination. There were certain alterations enriched but not specific to intracranial metastases, including frequent combined loss of tumor suppressors. We opted to perform whole genome sequencing for this study, as recent studies have revealed structural variants involving driver genes are identifiable in metastatic prostate cancer that may be missed through a targeted or whole exome approach^8^. Indeed, we identified not only *AR* enhancer amplification in the majority of brain metastases, but also a diversity of structural alterations that would not have been appreciated using an exome approach. We envision that these data of an additional 42 whole genomes of CRPC will contribute to the field’s growing understanding of the genomic landscape of metastatic prostate cancer at a broader scale.

Distinguishing mechanisms underlying tumor metastasis, bypassing the blood-brain barrier, and homing to the central nervous system is critical towards understanding the pathogenesis of brain metastases in prostate cancer. Equally important is the identification of factors that support tumor survival and adaptation in this vital organ, including interactions between tumor cells and neurons and the surrounding microenvironment. Our targeted gene expression analyses pointed to dysregulation of cytokeratins and cell adhesion molecules that may be important for homing to the brain microenvironment. Experimental models that recapitulate intracranial metastases in prostate cancer are currently lacking but may be feasible based on modeling in other cancer types such as potentially through intracardiac injection or other approaches. Our data provide a foundation to support additional preclinical studies to further characterize the pathogenesis of central nervous system metastases in prostate cancer.

A limitation of our study is the small sample size for molecular analysis due to patient selection and the requirement of tumor tissue, which is not feasible to obtain in most patients with intracranial metastases, as they are not often removed. Therefore, our whole genome analysis was limited to patients with a dominant lesion or limited metastases managed by metastatic resection or tumors obtained at time of autopsy. This therefore excluded patients with diffuse central nervous system involvement treated with radiotherapy, which is a classical metastatic pattern for many patients, including those with small cell neuroendocrine carcinoma. It not only remains challenging to obtain metastatic tissue from the cranium, but especially those matched with other anatomic sites of disease, to truly distinguish intracranial -specific patterns in individual patients. While our study was focused on genomic alterations, epigenetic alterations, metabolic, and other factors also contribute to therapy resistance in prostate cancer and may also influence patterns of metastatic spread.

## Methods

### Clinical cohort

Tumor and blood specimens were evaluated through protocols approved by the Weill Cornell Medicine (IRB #1610017620), Dana-Farber Cancer Institute (IRB #19–883), University of North Carolina (UNC IRB #08–0242) and Oregon Health Sciences (IRB #00019876) Institutional Review Boards (IRB #19–883, #1305013903). The study was conducted in accordance with the Declaration of Helsinki and the Good Clinical Practice guidelines. Patients with intracranial metastases were retrospectively identified through institutional databases and tumors were collected retrospectively or prospectively at the time of surgery/autopsy with written informed consent. Primary or non-brain metastatic tissue was evaluated in these same patients if tissue was available and obtained as part of clinical care. Additionally, patients with metastatic castration resistant prostate cancer (CRPC) who did not develop brain metastases were enrolled for profiling of their metastatic tumors (as a comparator) with written informed consent.

### Histology and immunohistochemistry

Tumor areas were annotated from frozen section or formalin fixed paraffin embedded (FFPE) H&E slides for macrodissection and DNA/RNA extraction. Immunohistochemistry for NKX3.1 (clone Rabbit Polyclonal, Biocare Medical), AR (clone F39.4.1, Biogenex), PSMA (clone 3E6, Dako), chromogranin (clone FH7, Leica), synaptophysin (clone 27G12, Leica), and ERG (EPR3864, Abcam) were performed on FFPE sections on a Leica Bond^TM^ system using the standard protocol F.

### Genomic sequencing

#### DNA sample preparation

Genomic DNA was extracted from frozen OCT-embedded tumors, macrodissected FFPE tumors, and blood specimens using Promega Maxwell 16 MDx per manufacturer’s instructions (Promega, Madison, WI). DNA quality and quantity were assessed using the Agilent Tapestation 4200 (Agilent Technologies) and Qubit Fluorometer (ThermoFisher), respectively. Sample libraries were prepared with different protocols, according to their RunID (see Supplementary Table 1).

#### WGS library preparation and sequencing, TruSeq Nano

Targeting 350 bp fragments (RUB_01399) or 450 bp fragments (PCCP_10601, PCCP_13816), whole genome sequencing (WGS) libraries were prepared using the Truseq DNA Nano Library Preparation Kit (Illumina 20015965) in accordance with the manufacturer’s instructions. Briefly, 100 ng of DNA was sheared using a Covaris LE220 sonicator (adaptive focused acoustics). DNA fragments underwent bead-based size selection and were subsequently end-repaired, adenylated, ligated to Illumina sequencing adapters, and amplified. Final libraries were quantified using the Qubit Fluorometer (Life Technologies) or Spectramax M2 (Molecular Devices) and Fragment Analyzer (Advanced Analytical) or Agilent 2100 BioAnalyzer. Libraries were sequenced on an Illumina HiSeqX sequencer using 2x150bp cycles.

#### WGS library preparation and sequencing, TruSeq PCR-free

Targeting 350 bp fragments (RUB_01212), whole genome sequencing (WGS) libraries were prepared using the Truseq DNA Nano Library Preparation Kit (Illumina 20015965) in accordance with the manufacturer’s instructions. Briefly, 1000 ng of DNA was sheared using a Covaris LE220 sonicator (adaptive focused acoustics). DNA fragments underwent bead-based size selection and were subsequently end-repaired, adenylated, and ligated to Illumina sequencing adapters. Final libraries were quantified using the ViiA 7 Real-Time PCR System (Applied Biosystems) and Fragment Analyzer (Advanced Analytical) or Agilent 2100 BioAnalyzer. Libraries were sequenced on an Illumina HiSeq 2000 sequencer using 2x100bp cycles.

#### WGS library preparation and sequencing, KAPA Hyper PCR Plus

Targeting 500 bp fragments (KAU_13605, KAU_13666, KIM_14128), whole genome sequencing (WGS) libraries were prepared using the KAPA Hyper Library Preparation Kit (KAPABiosystems KK8502, KK8504) in accordance with the manufacturer’s instructions. Briefly, 200 ng of DNA was sheared using a Covaris LE220 sonicator (adaptive focused acoustics). DNA fragments were end-repaired, adenylated, ligated to Illumina sequencing adapters, underwent bead-based size selection and were amplified. Final libraries were quantified using the Qubit Fluorometer (Life Technologies) or Spectramax M2 (Molecular Devices) and Fragment Analyzer (Advanced Analytical) or Agilent 2100 BioAnalyzer. Libraries were sequenced on an Illumina Novaseq6000 sequencer using 2x150bp cycles.

### Whole genome sequencing processing and analysis

#### Preprocessing

Sequencing reads for the tumor and normal samples were aligned to the GRCh38 reference using BWA-MEM (v0.7.15)^21^. NYGC’s ShortAlignmentMarking (v2.1) was used to mark short reads as unaligned^22^ (https://github.com/nygenome/nygc-short-alignment-marking). GATK (v4.1.0)^23^ FixMateInformation was run to verify and fix mate-pair information, followed by Novosort (v1.03.01) markDuplicates to merge individual lane BAM files into a single BAM file per sample. Duplicates were then sorted and marked, and GATK’s base quality score recalibration (BQSR) was performed.

#### Somatic variant calling

The tumor and normal bam files were processed through NYGC’s variant calling pipeline^24^, which consists of MuTect2 (GATK v4.0.5.1)^25^, Strelka2 (v2.9.3)^26^ and Lancet (v1.0.7)^27^ for calling Single Nucleotide Variants (SNVs) and short Insertion-or-Deletion (Indels), SvABA (v0.2.1)^28^ for calling Indels and Structural variants (SVs), Manta (v1.4.0)^29^ and Lumpy (v0.2.13)^30^ for calling SVs. Manta also outputs a candidate set of Indels which was provided as input to Strelka2. Lancet is only run on the exonic part of the genome. It is also run on the +/− 250nt regions around nonexonic variants that are called by only one of the other callers, to add confidence to such variants. Small SVs called by Manta are also used to add confidence to the indel calls.

Variant calls were merged by variant type (SNVs, Multi-Nucleotide Variants (MNVs), Indels and SVs). MuTect2 and Lancet call MNVs, however Strelka2 does not, and it also does not provide any phasing information. To merge such variants across callers, we first split the MNVs called by MuTect2 and Lancet to SNVs, and then merged the SNV callsets across the different callers. 3 If the caller support for each SNV in a MNV was the same, we merged them back to MNVs. Otherwise those are represented as individual SNVs in the final callset. Lancet and Manta are the only tools that can call deletion-insertion events. Other tools may represent the same event as separate yet adjacent indel and/or SNV variants. Such events are relatively less frequent, and difficult to merge. We therefore did not merge these calls with SNV and Indel calls from other callers. All SVs below 500 bp were excluded and the rest merged across callers using bedtools^31^ pairtopair (requiring slop of 300 bp, same strand orientation, and 50% reciprocal overlap).

#### Somatic variant annotation and filtering

SNVs and Indels were annotated with Ensembl as well as databases such as COSMIC (v86)^32^, 1000Genomes (Phase3)^33^, ClinVar (201706)^34^, PolyPhen (v2.2.2)^35^, SIFT (v5.2.2)^36^, FATHMM (v2.1)^37^, gnomAD (r2.0.1)^38^ and dbSNP (v150)^39^ using Variant Effect Predictor (v93.2)^40^.

All predicted SVs were annotated with germline variants by overlapping with known variants in 1000 Genomes and Database of Genomic Variants (DGV)^41^. Cancer-specific annotation included overlap with genes from Ensembl^42^ and Cancer Gene Census in COSMIC, and potential effect on gene structure (e.g. disruptive, intronic, intergenic). If a predicted SV disrupted two genes and strand orientations are compatible, it was annotated as a putative gene fusion candidate. Further annotations include sequence features within breakpoint flanking regions, e.g. mappability, simple repeat content and segmental duplications.

For SNVs, Indels, and SVs, we used an in-house panel of normals (PON) to filter putative artifacts. Somatic SNVs and Indels were filtered out if they were found in more than two or more individuals in our PON. To filter our somatic SV callset, we identified calls in our PON using bedtools pairtopair (requiring slop of 300 bp, same strand orientation, and 50% reciprocal overlap), and filtered those SVs found in two or more individuals in our PON. In addition to the PON filtering, we removed SNVs and Indels that have minor allele frequency (MAF) of 1% or higher in either 1000 Genomes Phase 3 or gnomAD (r2.0.1)^38^, and SVs that overlap DGV, 1000Genomes Phase 3, or gnomAD SV^43^.

As our callset was generated by merging calls across callers, and each of them reported different allele counts, we report final chosen allele counts for SNVs and indels. For SNVs, and for indels less than 10nt in length, these were computed as the number of unique read-pairs supporting each allele using the pileup method, with minimum mapping quality and base quality thresholds of 10 each. For larger indels and complex (deletion-insertion) events, we chose the final allele counts reported by the individual callers Strelka2, MuTect2, Lancet, in that order. For indels larger than 10nt that are only called by SvABA, we do not report final allele counts and allele frequencies because SvABA does not report the reference allele count, making it difficult to estimate the variant allele frequency. We then used these final chosen allele counts and frequencies to filter the somatic callset. Specifically, we filtered any variant for which the variant allele frequency (VAF) in the tumor sample is less than 0.0001, if the VAF in the normal sample was greater than 0.2, or if the sequencing depth at the position was less than 2 in either the tumor sample or the normal sample. We also filtered variants for which the VAF in the normal sample is greater than the VAF in the tumor sample.

For our final SNV and Indel callset, we retained calls that passed the above-mentioned filters, and were either called by two or more variant callers, or called by one caller and also seen in the Lancet validation calls or in the Manta SV calls. For patients with multiple samples, a union of somatic SNVs and Indels across all the patient’s samples was generated. Pileup (0.15.0)^44^ was then run on tumor and normal bam files to compute the read support for variants present in the union that were missing from each sample’s callset. Variants with allele frequency greater than 0 were then rescued.

For our final SV callset, we retained calls that passed the above-mentioned filters, and were either called by 2 or more variant callers, or called by Manta or Lumpy with either additional support from a nearby CNV changepoint, or split-read support from SplazerS (Emde et al., Bioinformatics 2012). An SV is considered supported by SplazerS if it found at least 3 split-reads in the tumor only. Nearby CNV changepoints were determined by overlapping BIC-Seq2 calls with the SV callset using bedtools closest. An SV was considered to be supported by a CNV changepoint if the breakpoint of the CNV is within 1000 bp of an SV breakpoint. For cases with multiple samples, read support for the union of SVs was calculated, and SVs with read support greater than 0 were rescued.

#### Copy number and complex structural variants

For each sample, GC content and mappability-corrected read depth data was computed in 1Kbp bins using fragCounter^45^. The read depth data was then corrected for systematic artifacts using dryclean^46^ by building a PON from the normal samples used in this study and applying to all tumor samples. Purity and ploidy were estimated for each sample by running AscatNGS^47^ and Sequenza^48^, and manually reviewing to select the most accurate estimate. Junction-balanced genome graphs with genomic interval and junction integer copy number were generated by running Jabba^49^ with the SV callset, manually curated purity and ploidy estimates, dryclean-corrected tumor read depth data, and B-allele frequency data as input. gGnome^50^ was then used to call simple and complex structural variants.

Focal copy number variants (<= 3MB) were determined relative to a sample’s copy-neutral state, as defined by ploidy. For samples with an intermediate average ploidy (fractional value between 0.4 and 0.6, e.g. 3.5), neutral copy state was set as the closest two integer values (e.g. for a ploidy of 3.5, neutral copy states would be 3 and 4). Otherwise, the neutral copy state was set as the rounded ploidy. Events above the neutral copy number were classified as gains, and those more than double ploidy were classified as amplifications. Conversely, events below the neutral copy number were classified as deletions. Events with a copy number of 0 were classified as losses.

#### AR enhancer coordinates

The AR enhancer coordinates described in^51^ (GRCh37: 66,100,000–66,155,000) were lifted over from GRCh37 to GRCh38 (GRCh38: chrX:66,880,158–66,935,158) using the UCSC LiftOver tool^52^.

#### Mutation timing

The MutationTimeR R package^53^ was run using somatic SNVs and INDELs, allele-specific copy number output from JaBba, patient gender information, and sample purity estimates. Parameter n.boot was set to 200. MutationTimer infers a multiplicity for each mutation, and assigns a timing based on the multiplicity and the allele-specific copy number configuration at that locus. Using MutationTimer multiplicities, cancer cell fraction was computed as follows^54^:

$$CCF=\frac{f}{n\rho }(\rho {N}_{T}+{N}_{N}(1-\rho ))$$

Where n is the mutation multiplicity, p is the tumor purity, f is the mutation VAF, and N_T_ is the tumor total copy number at the mutation locus, and N_N_ is the normal total copy number at the mutation locus.

#### Fraction of genome altered

The fraction of genome altered (FGA) was calculated as the proportion of autosomes not in the previously defined copy-neutral state.

### Nanostring profiling

Tumor mRNA was extracted from scraped unstained slides using the Promega Maxwell® 16 LEV RNA FFPE Purification Kit (Cat. #AS1260) or QIAGEN RNeasy FFPE Kit (Cat. #73504). RNA quality control was performed with the Agilent 2100 Bioanalyzer system by annotating total RNA concentration and percentage of RNA greater than 300 nucleotides (nt) in length. At least 100 ng of RNA greater than 300nt in length was required for downstream analysis, and the exact amount of input RNA was proportionally increased according to the level of degradation. Samples were run on the NanoString nCounter® Analysis System according to the manufacturer directions. A 361 custom gene panel was developed based on their known and potential roles in prostate cancer progression, including AR and AR signaling genes^55^, the AR V7 splice variant, EMT/plasticity and neuroendocrine prostate cancer associated genes^56^, cell cycle, WNT, PI3K/AKT pathway genes, TMPRSS2-ERG fusion transcript, and control and housekeeper genes. Nanostring raw counts were normalized by a RUVSeq-based process^57^, which performs both upper quartile normalization^58^ and normalization with RUVg^59^ to estimate RUV factors using the endogenous housekeeping genes. DESeq2^60^ package was applied to determine differentially expressed genes. For comparisons, Benjamini-Hochberg was performed for multiple-testing correction. A gene was considered significant if the adjusted *p*-value was less than 0.05 and the logFC was more than 1 or 1.5.

### Reporting summary

Further information on research design is available in the Nature Research Reporting Summary linked to this article.

### Supplementary information

Supplementary Figures

Supplementary Table 1

Supplementary Table 2

Reporting Summary

## Data Availability

Sequencing data is accessible via dbGaP (accession number phs003357.v1.p1).

## Code availability

The open source software packages used to conduct data processing and analysis were: BWA-MEM (v0.7.15, https://github.com/lh3/bwa), ShortAlignmentMarking (v2.1, https://github.com/nygenome/nygc-short-alignment-marking), GATK (v4.1.0, https://gatk.broadinstitute.org/hc/en-us), Novosort (v1.03.01, https://www.novocraft.com/products/novosort), MuTect2 (GATK v4.0.5.1, https://gatk.broadinstitute.org/hc/en-us/articles/360036463432-Mutect2), Strelka2 (v2.9.3, https://github.com/Illumina/strelka), Lancet (v1.0.7, https://github.com/nygenome/lancet), SvABA (v0.2.1, https://github.com/walaj/svaba), Manta (v1.4.0, https://github.com/Illumina/manta), Lumpy (v0.2.13, https://github.com/arq5x/lumpy-sv), bedtools (2.27.1, https://github.com/arq5x/bedtools2), Ensembl Variant Effect Predictor (v93.2, https://github.com/Ensembl/ensembl-vep), fragCounter (https://github.com/mskilab-org/fragCounter), dryclean (https://github.com/mskilab-org/dryclean), AscatNGS (v4.2.1, https://github.com/cancerit/ascatNgs), Sequenza (v3.0.0, https://bitbucket.org/sequenzatools/sequenza/src/master/), JaBbA (v1.1, https://github.com/mskilab-org/JaBbA), gGnome (v1.0, https://github.com/mskilab-org/gGnome), MutationTimeR (v1.00.2, https://github.com/gerstung-lab/MutationTimeR), RUVSeq (https://bioconductor.org/packages/release/bioc/html/RUVSeq.html), and DESeq2 (https://bioconductor.org/packages/release/bioc/html/DESeq2.html). Additional software packages for figure generation are listed in the GitHub repository associated with this manuscript. Statistical analyses were performed using R 3.6.1. Custom analysis scripts and scripts to reproduce figures are available at: https://github.com/nygenome/ProstateBrainMet_WGS_paper_figures.
