# Author Correction: Evolution of structural rearrangements in prostate cancer intracranial metastases

**DOI:** 10.1038/s41698-023-00469-7

**Published:** 2023-11-02

**Authors:** Francesca Khani, William F. Hooper, Xiaofei Wang, Timothy R. Chu, Minita Shah, Lara Winterkorn, Michael Sigouros, Vincenza Conteduca, David Pisapia, Sara Wobker, Sydney Walker, Julie N. Graff, Brian Robinson, Juan Miguel Mosquera, Andrea Sboner, Olivier Elemento, Nicolas Robine, Himisha Beltran

**Affiliations:** 1 Department of Pathology and Laboratory Medicine, Weill Cornell Medicine, New York, NY USA; 2 Englander Institute for Precision Medicine, Weill Cornell Medicine, New York, NY USA; 3 New York Genome Center, New York, NY USA; 4 Department of Medical and Surgical Sciences, Unit of Medical Oncology and Biomolecular Therapy, University of Foggia, Policlinico Riuniti, Foggia, Italy; 5 Department of Pathology and Laboratory Medicine, UNC Chapel Hill, Chapel Hill, NC USA; 6 Department of Medical Oncology, Oregon Health Sciences University, Portland, OR USA; 7 The HRH Prince Alwaleed Bin Talal Bin Abdulaziz Alsaud Institute for Computational Biomedicine, Weill Cornell Medicine, New York, NY USA; 8 Meyer Cancer Center, Weill Cornell Medicine, New York, NY USA; 9 Department of Physiology and Biophysics, Weill Cornell Medicine, New York, NY USA; 10 Department of Medical Oncology, Dana-Farber Cancer Institute, Boston, MA USA

**Keywords:** Prostate cancer, Cancer genomics

Correction to: *npj Precision Oncology* 10.1038/s41698-023-00435-3, published online 13 September 2023

The original Abstract incorrectly stated this was the first whole genome sequencing (WGS) study of prostate cancer intracranial metastases. There were two subdural metastases included in a prior prostate cancer WGS landscape study by Gundhem et al., 2015. The Abstract has been revised to correct this statement. The work of Gundhem et al. is now cited in the Introduction and the Reference list has been updated to include this citation.

