# Supplementary material for: Evolution of structural rearrangements in prostate cancer intracranial metastases
Source: NPJ Precis Oncol. 2023 Sep 13;7:91. doi: 10.1038/s41698-023-00435-3 (PMC10499931; doi:10.1038/s41698-023-00435-3)
Supplement: Supplementary file 1 — Supplementary Figures [file 41698_2023_435_MOESM1_ESM.pdf]

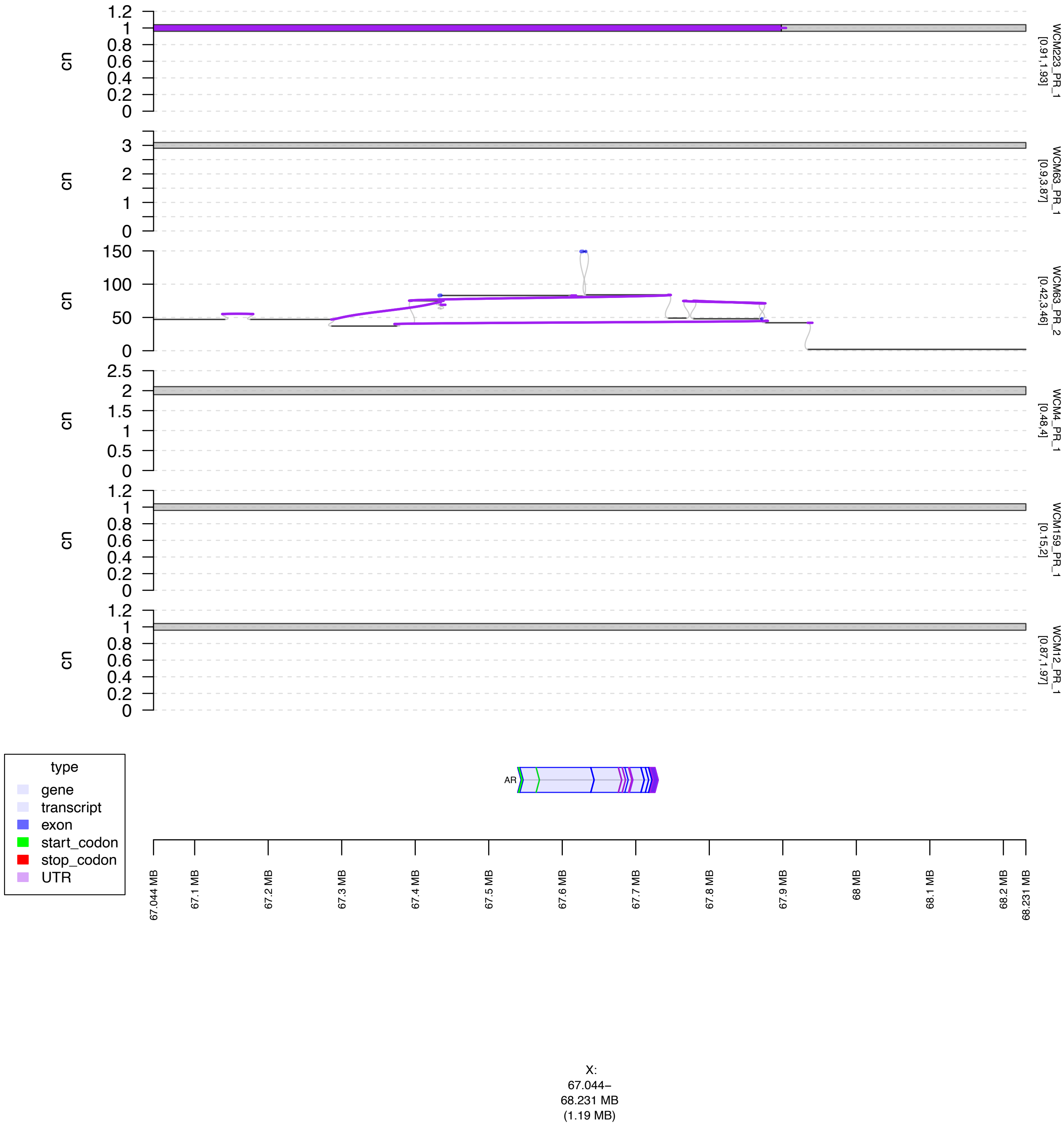

Supplementary Figure 1A: Complex structural variants at the AR loci in prostate samples. The horizontal bars represent genomic regions, and their height represents their clonal copy number. Reference adjacencies are indicated by thin grey lines connecting segments. Non-reference adjacencies (i.e., structural variant breakpoints), as well as any genomic regions that are part of a JaBbA event are colored purple. The location of AR and is indicated at the bottom of the panel. Sample purity and ploidy are indicated below the sample names on the right side of the plot.

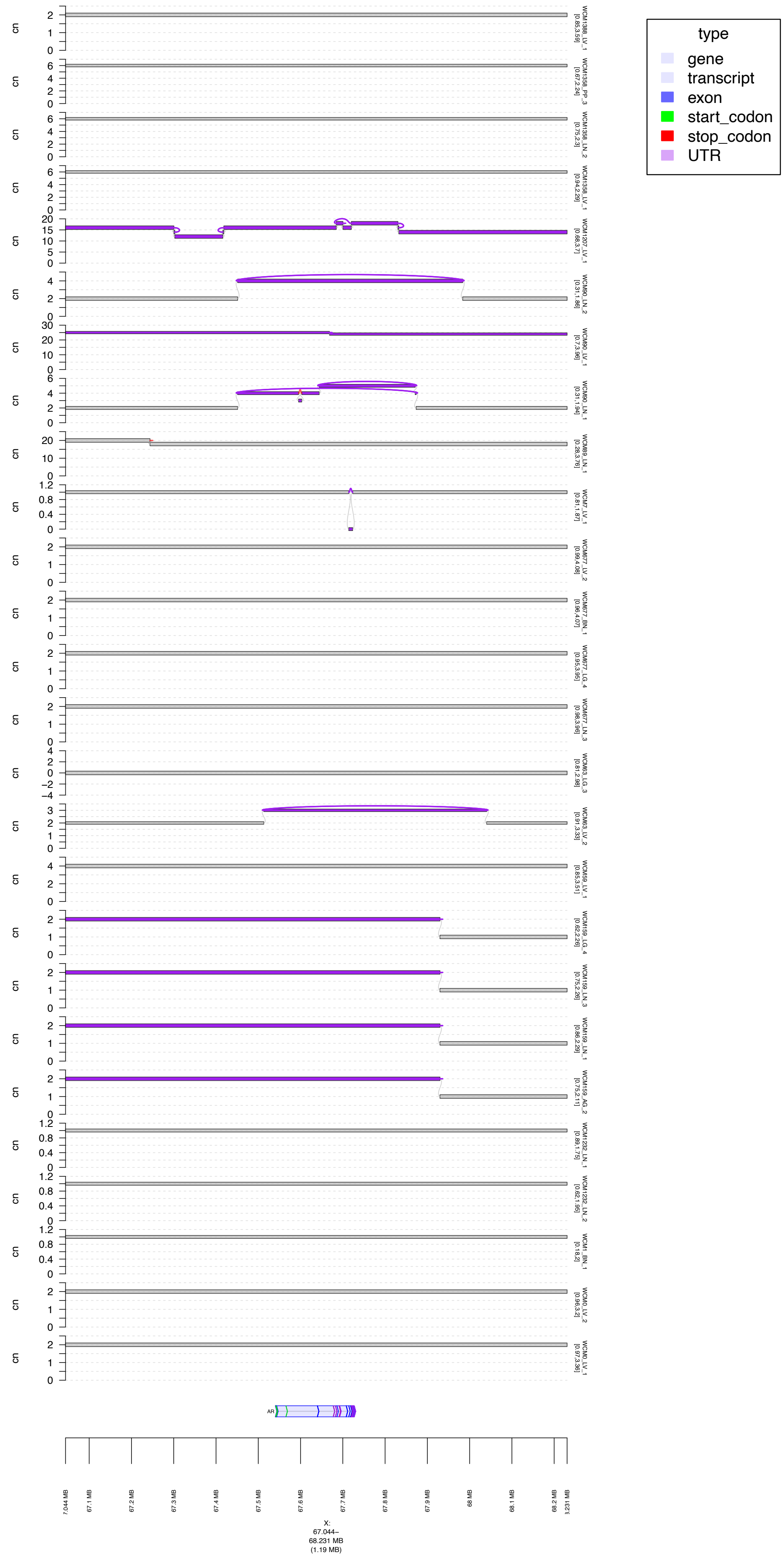
type
gene
transcript
exon
start_codon
stop_codon
UTR
cn
AR
X:
67.044–
68.231 MB
(1.19 MB)

Supplementary Figure 1B: Complex structural variants at the AR loci in non-brain metastatic samples. The horizontal bars represent genomic regions, and their height represents their clonal copy number. Reference adjacencies are indicated by thin grey lines connecting segments. Non-reference adjacencies (i.e., structural variant breakpoints), as well as any genomic regions that are part of a JaBbA event are colored purple. The location of AR and is indicated at the bottom of the panel. Sample purity and ploidy are indicated below the sample names on the right side of the plot.

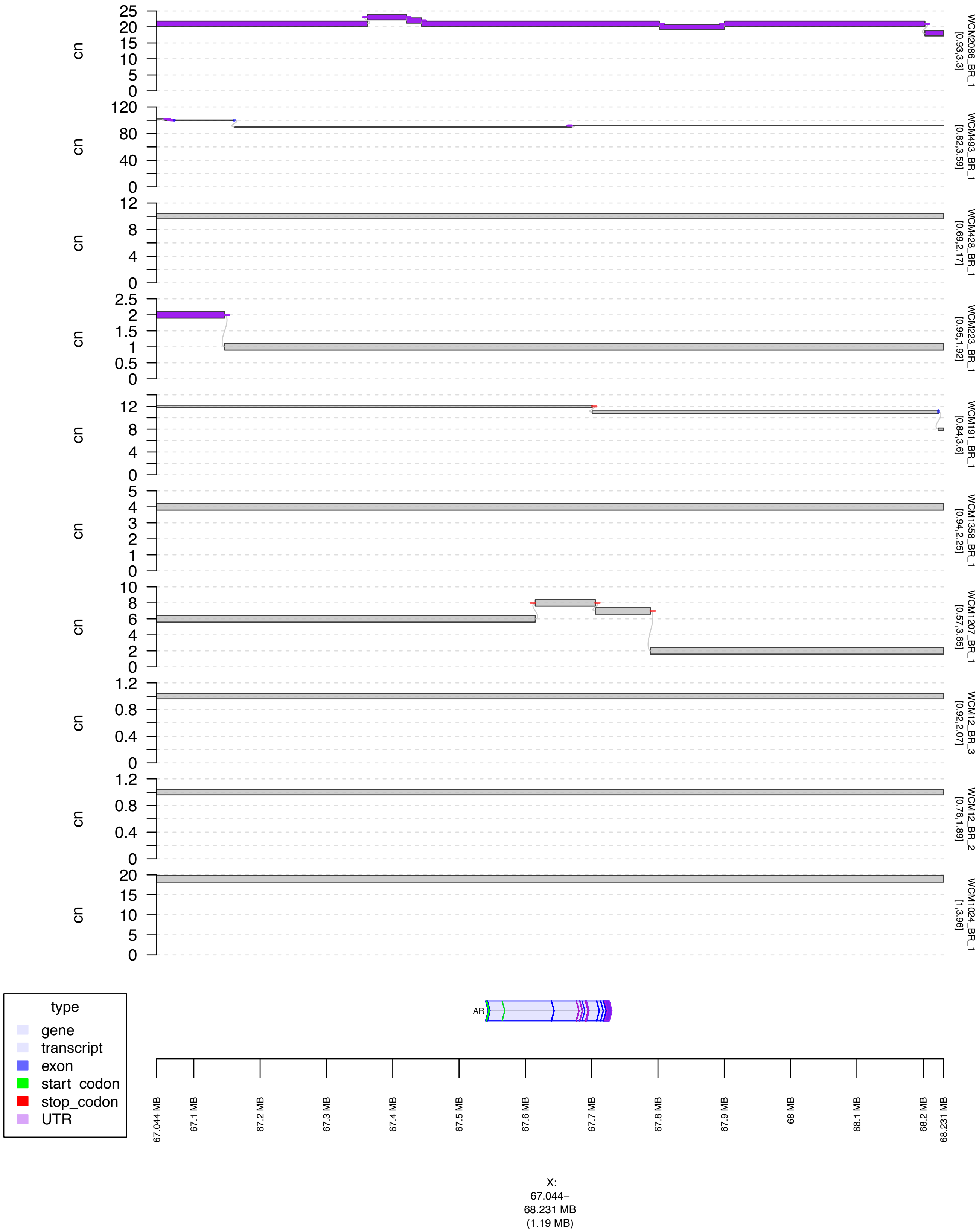

Supplementary Figure 1C: Complex structural variants at the AR loci in brain metastatic samples. The horizontal bars represent genomic regions, and their height represents their clonal copy number. Reference adjacencies are indicated by thin grey lines connecting segments. Non-reference adjacencies (i.e., structural variant breakpoints), as well as any genomic regions that are part of a JaBbA event are colored purple. The location of AR and is indicated at the bottom of the panel. Sample purity and ploidy are indicated below the sample names on the right side of the plot.

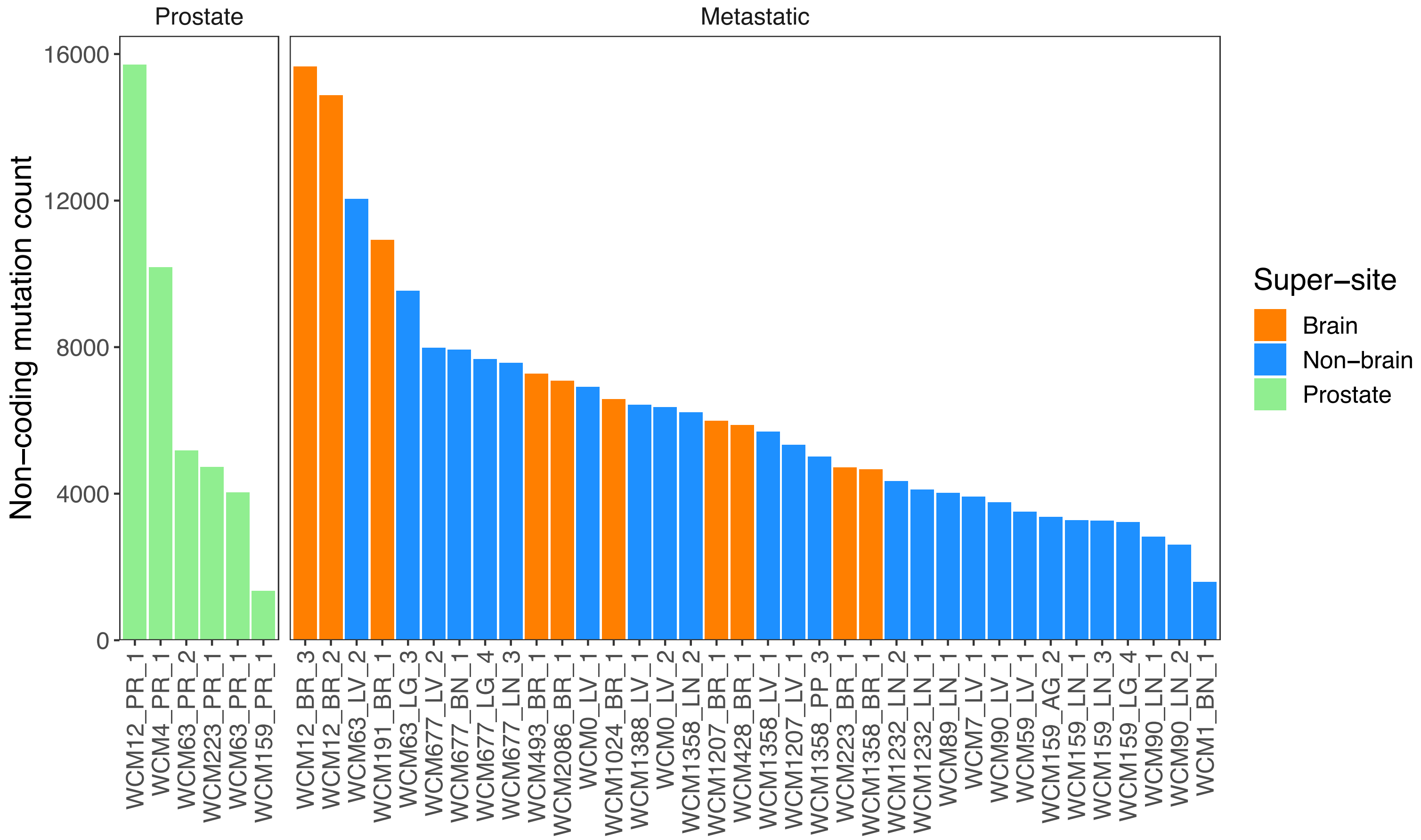

Supplementary Figure 2: Noncoding mutation burden. The vertical bars represent the number of non-coding mutations detected for each sample. Samples are sorted within prostate and metastatic groupings by total non-coding mutation count, and each bar is colored by super-site.

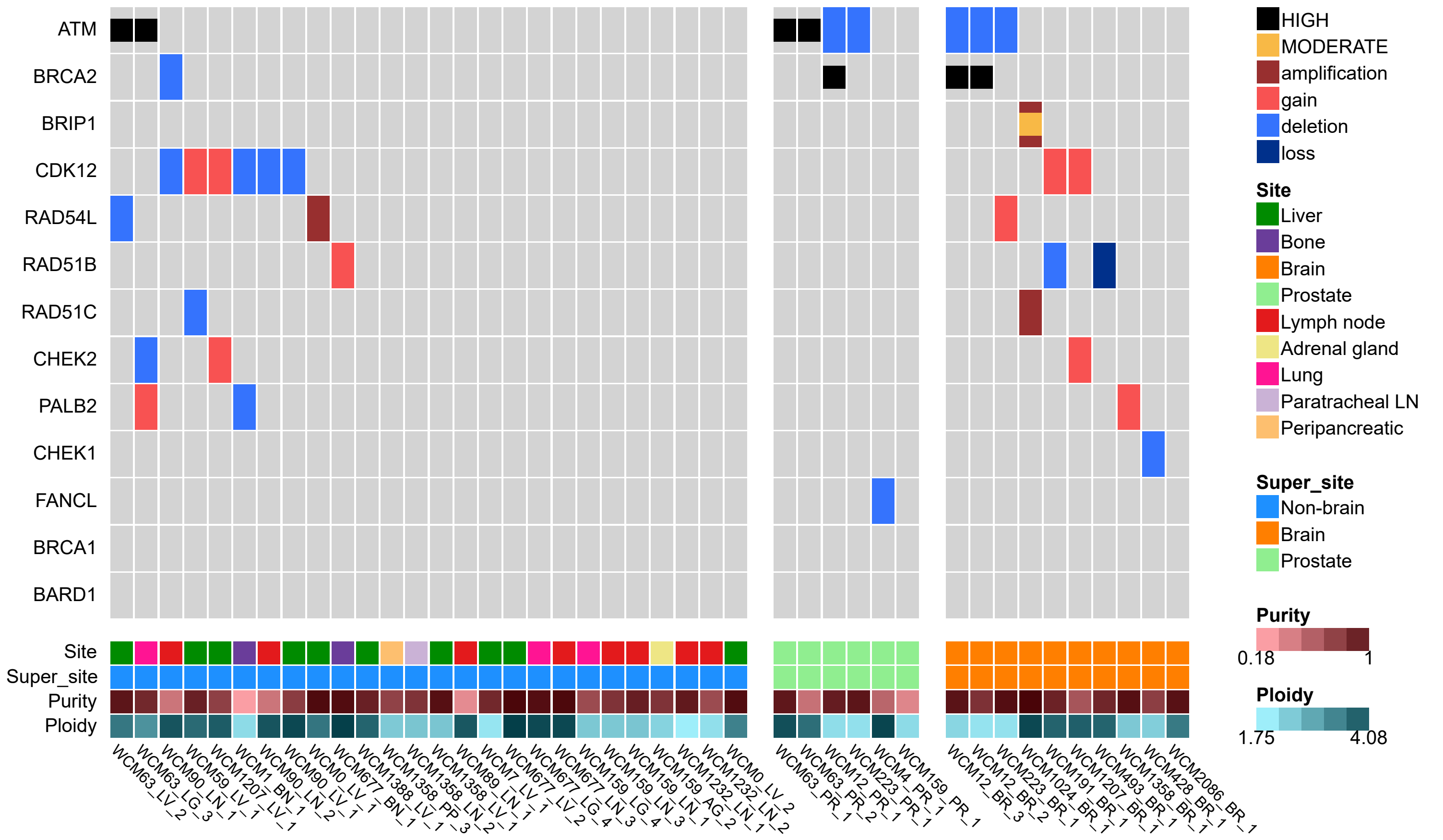

Supplementary Figure 3: Oncoprint of HRR genes. Oncoprint of focal copy changes, as well as moderate and high-impact SNV/INDELS, sorted by variant prevalence across the cohort and color coded by variant category. Copy number variants larger than 3MB in length are excluded from the figure.

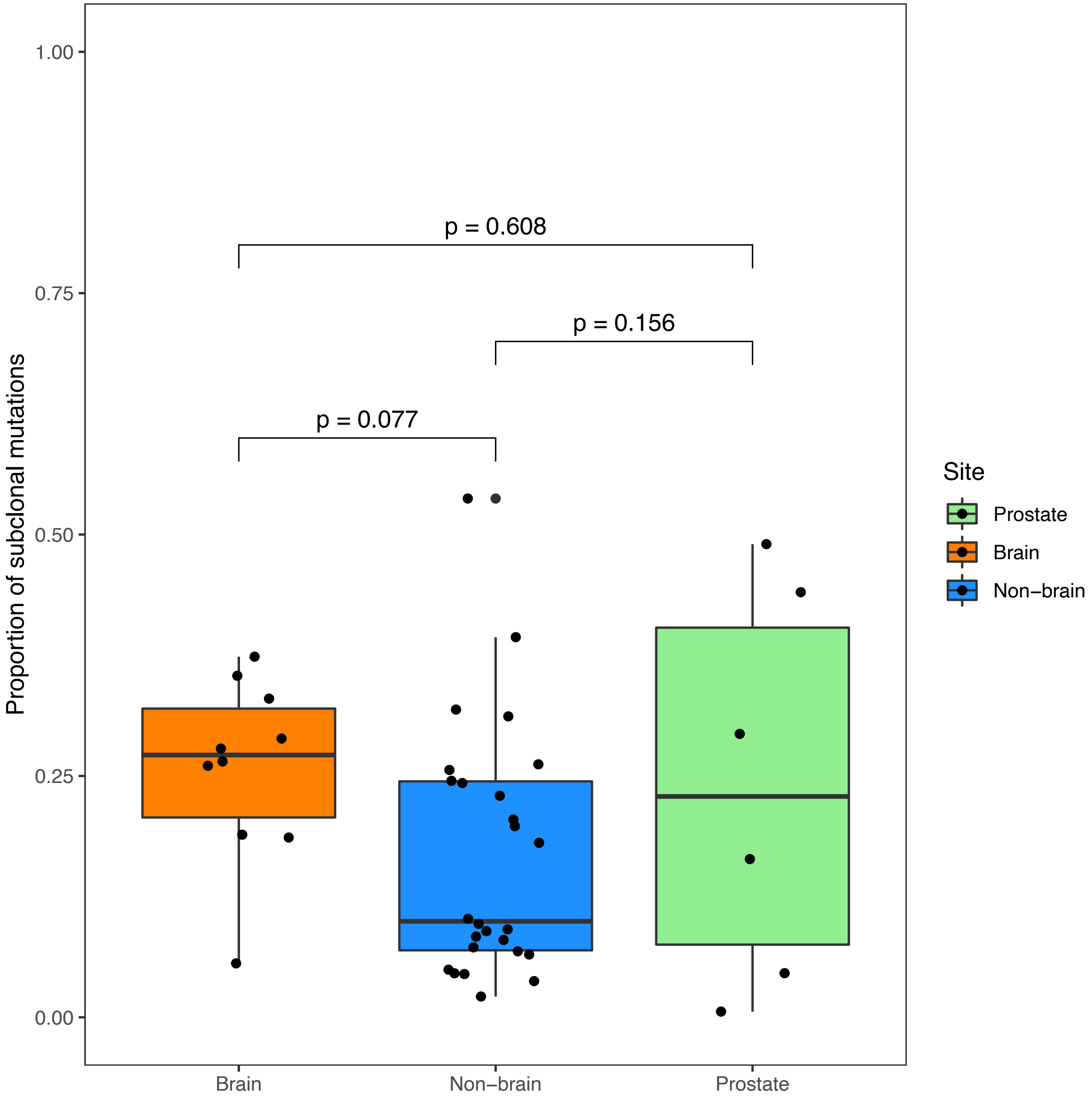

Supplementary Figure 4: Proportion of subclonal mutations in prostate (green), brain metastasis (orange) and non-brain metastasis (blue). Each point represents a sample. The horizontal line in each box represents the median for each group. The bottom and top of each box represent the first and third quartiles, respectively. The vertical lines extend to the values no farther than 1.5 times the interquartile range. Groups were compared using an ANCOVA test, with sample purity as a covariate.

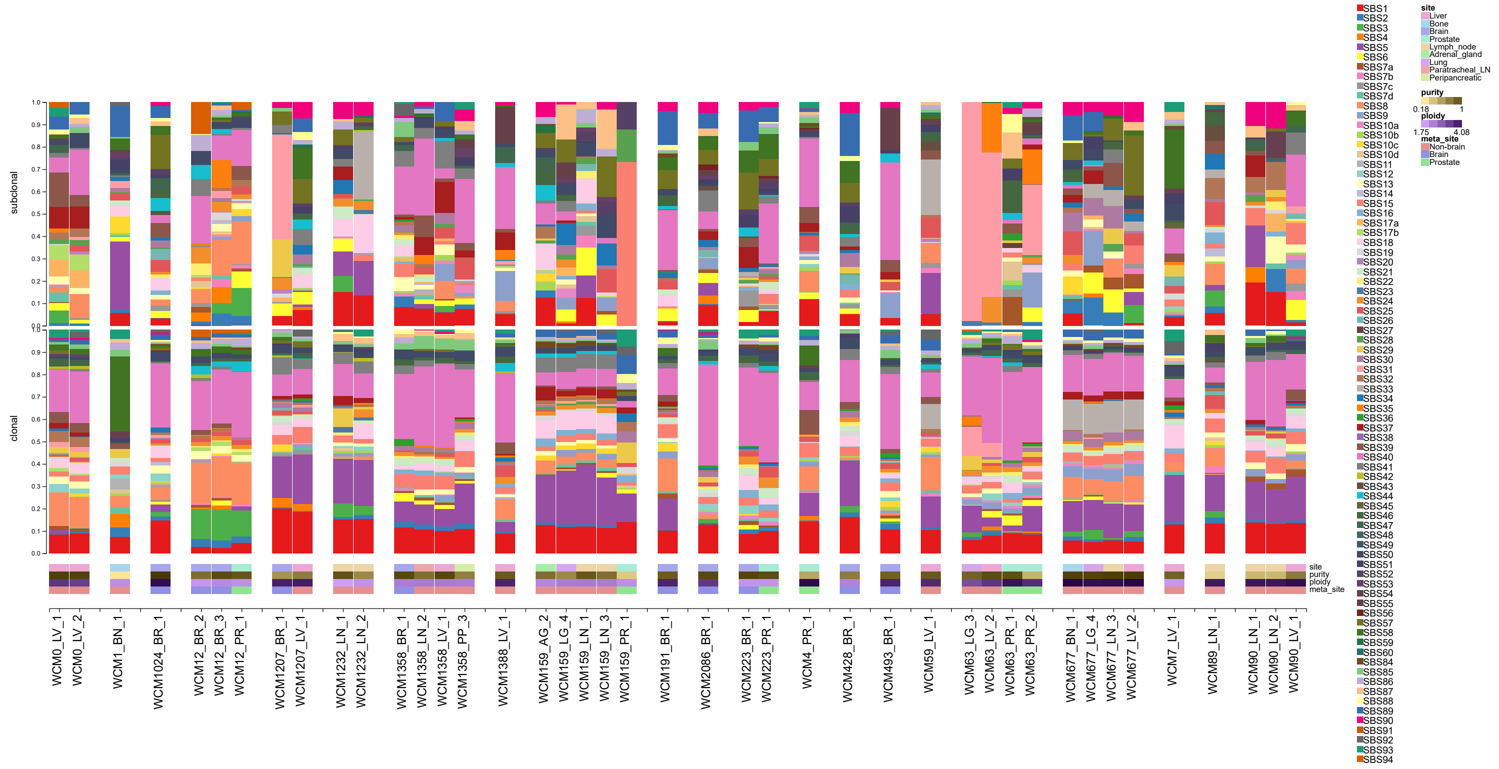

Supplementary Figure 5: Mutational signatures of clonal and subclonal mutations, as estimated by deconstructSigs. The top panel represents the relative signature contributions for subclonal mutations, and the bottom panel represents clonal mutations.

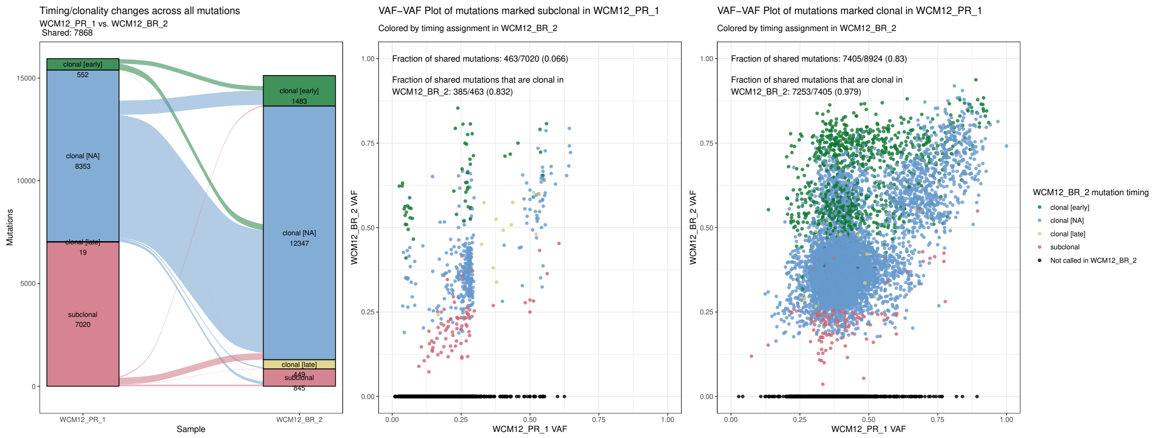
Timing/clonality changes across all mutations
WCM12_PR_1 vs. WCM12_BR_2
Shared: 7868
Mutations
Sample
WCM12_PR_1
WCM12_BR_2
clonal [early] 552
clonal [NA] 8353
clonal [late] 19
subclonal 7020
clonal [early] 1483
clonal [NA] 12347
clonal [late]
subclonal 845
VAF−VAF Plot of mutations marked subclonal in WCM12_PR_1
Colored by timing assignment in WCM12_BR_2
Fraction of shared mutations: 463/7020 (0.066)
Fraction of shared mutations that are clonal in WCM12_BR_2: 385/463 (0.832)
WCM12_BR_2 VAF
WCM12_PR_1 VAF
VAF−VAF Plot of mutations marked clonal in WCM12_PR_1
Colored by timing assignment in WCM12_BR_2
Fraction of shared mutations: 7405/8924 (0.83)
Fraction of shared mutations that are clonal in WCM12_BR_2: 7253/7405 (0.979)
WCM12_BR_2 mutation timing
clonal [early]
clonal [NA]
clonal [late]
subclonal
Not called in WCM12_BR_2

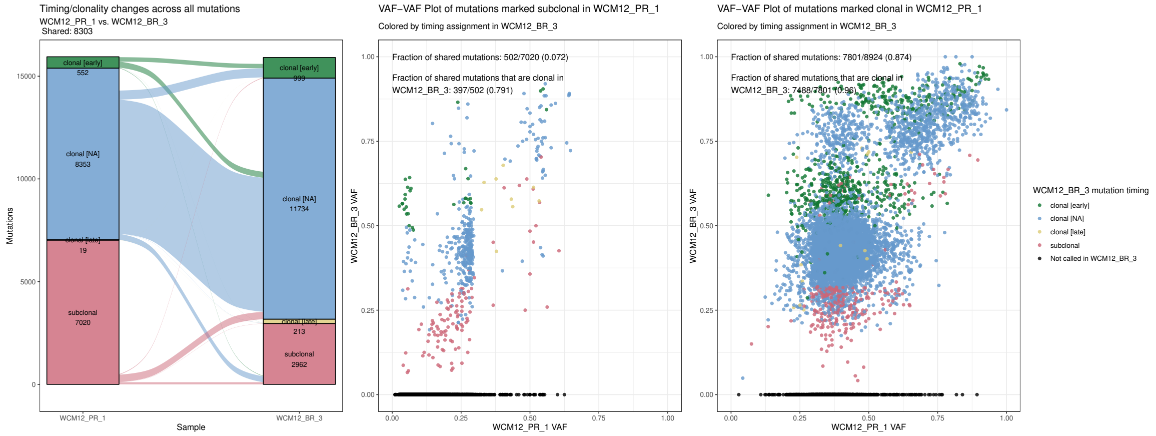
Timing/clonality changes across all mutations
WCM12_PR_1 vs. WCM12_BR_3
Shared: 8303
Mutations
Sample
WCM12_PR_1
WCM12_BR_3
clonal [early] 552
clonal [NA] 8353
clonal [late] 19
subclonal 7020
clonal [early] 939
clonal [NA] 11734
clonal [late] 213
subclonal 2962
VAF−VAF Plot of mutations marked subclonal in WCM12_PR_1
Colored by timing assignment in WCM12_BR_3
Fraction of shared mutations: 502/7020 (0.072)
Fraction of shared mutations that are clonal in WCM12_BR_3: 397/502 (0.791)
WCM12_BR_3 VAF
WCM12_PR_1 VAF
VAF−VAF Plot of mutations marked clonal in WCM12_PR_1
Colored by timing assignment in WCM12_BR_3
Fraction of shared mutations: 7801/8924 (0.874)
Fraction of shared mutations that are clonal in WCM12_BR_3: 7488/7801 (0.96)
WCM12_BR_3 mutation timing
clonal [early]
clonal [NA]
clonal [late]
subclonal
Not called in WCM12_BR_3

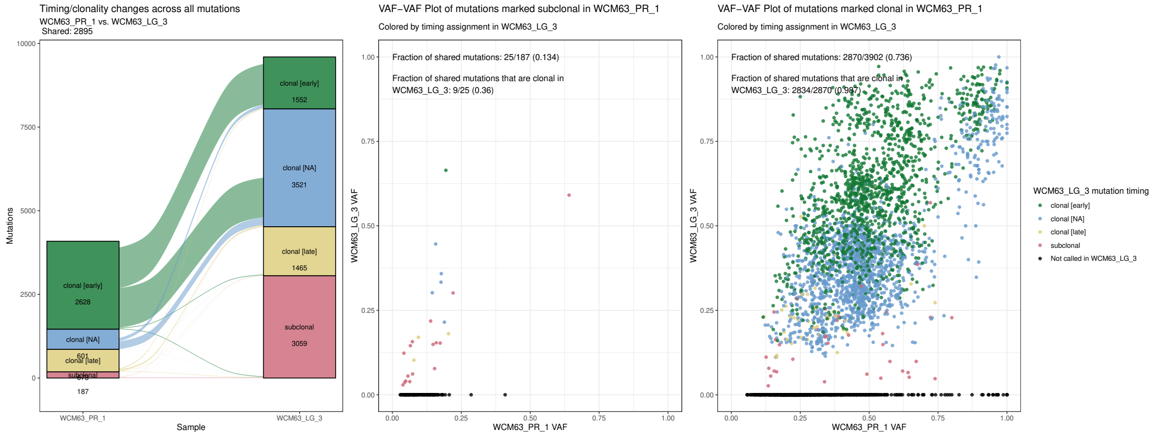
Timing/clonality changes across all mutations
WCM63_PR_1 vs. WCM63_LG_3
Shared: 2895
Mutations
Sample
WCM63_PR_1
WCM63_LG_3
clonal [early] 2628
clonal [NA] 601
clonal [late]
subclonal 187
clonal [early] 1552
clonal [NA] 3521
clonal [late] 1465
subclonal 3059
VAF−VAF Plot of mutations marked subclonal in WCM63_PR_1
Colored by timing assignment in WCM63_LG_3
Fraction of shared mutations: 25/187 (0.134)
Fraction of shared mutations that are clonal in WCM63_LG_3: 9/25 (0.36)
WCM63_LG_3 VAF
WCM63_PR_1 VAF
VAF−VAF Plot of mutations marked clonal in WCM63_PR_1
Colored by timing assignment in WCM63_LG_3
Fraction of shared mutations: 2870/3902 (0.736)
Fraction of shared mutations that are clonal in WCM63_LG_3: 2834/2870 (0.987)
WCM63_LG_3 mutation timing
clonal [early]
clonal [NA]
clonal [late]
subclonal
Not called in WCM63_LG_3

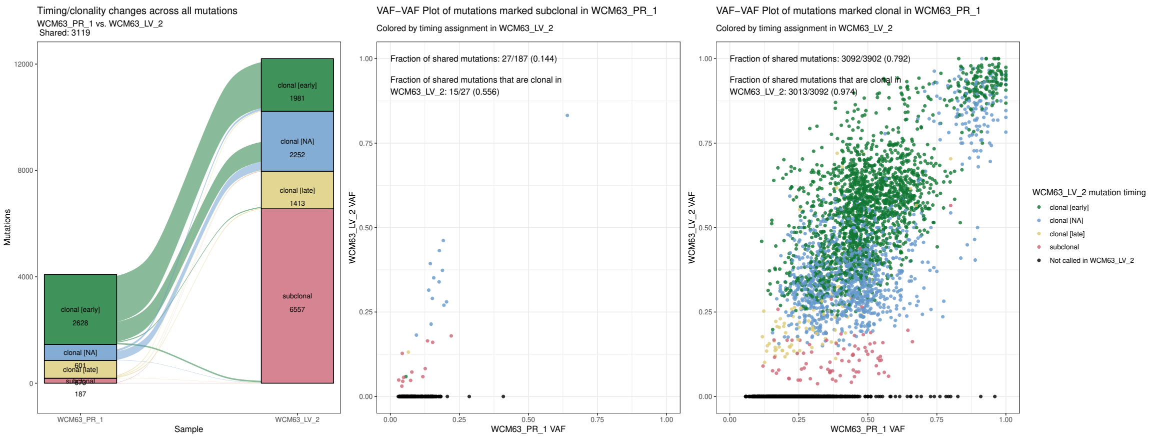
Timing/clonality changes across all mutations
WCM63_PR_1 vs. WCM63_LV_2
Shared: 3119
Mutations
Sample
WCM63_PR_1
WCM63_LV_2
clonal [early] 2628
clonal [NA]
clonal [late]
subclonal
187
clonal [early] 1981
clonal [NA] 2252
clonal [late] 1413
subclonal 6557
VAF−VAF Plot of mutations marked subclonal in WCM63_PR_1
Colored by timing assignment in WCM63_LV_2
Fraction of shared mutations: 27/187 (0.144)
Fraction of shared mutations that are clonal in WCM63_LV_2: 15/27 (0.556)
WCM63_LV_2 VAF
WCM63_PR_1 VAF
VAF−VAF Plot of mutations marked clonal in WCM63_PR_1
Colored by timing assignment in WCM63_LV_2
Fraction of shared mutations: 3092/3902 (0.792)
Fraction of shared mutations that are clonal in WCM63_LV_2: 3013/3092 (0.974)
WCM63_LV_2 mutation timing
clonal [early]
clonal [NA]
clonal [late]
subclonal
Not called in WCM63_LV_2

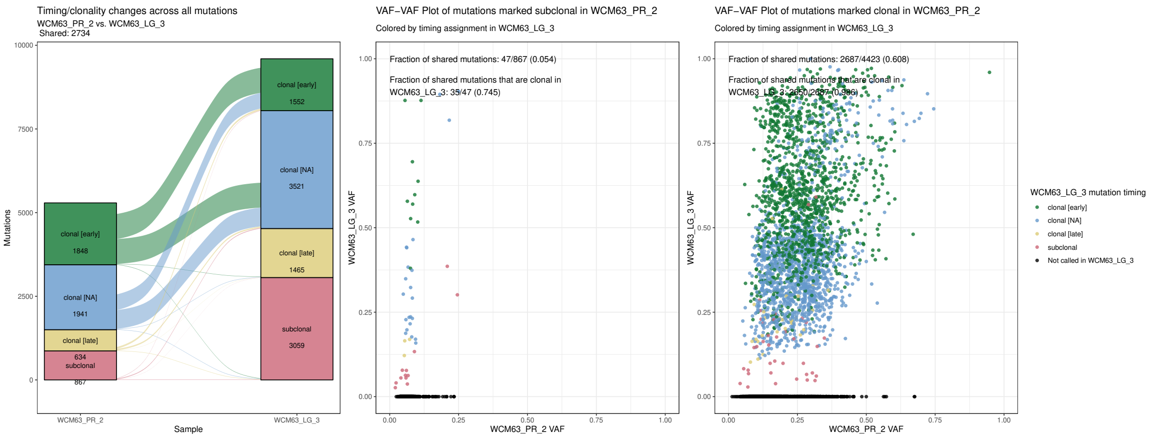
Timing/clonality changes across all mutations
WCM63_PR_2 vs. WCM63_LG_3
Shared: 2734
Mutations
Sample
WCM63_PR_2
WCM63_LG_3
clonal [early] 1848
clonal [NA] 1941
clonal [late] 634
subclonal 867
clonal [early] 1552
clonal [NA] 3521
clonal [late] 1465
subclonal 3059
VAF−VAF Plot of mutations marked subclonal in WCM63_PR_2
Colored by timing assignment in WCM63_LG_3
Fraction of shared mutations: 47/867 (0.054)
Fraction of shared mutations that are clonal in WCM63_LG_3: 35/47 (0.745)
WCM63_LG_3 VAF
WCM63_PR_2 VAF
VAF−VAF Plot of mutations marked clonal in WCM63_PR_2
Colored by timing assignment in WCM63_LG_3
Fraction of shared mutations: 2687/4423 (0.608)
WCM63_LG_3 mutation timing
clonal [early]
clonal [NA]
clonal [late]
subclonal
Not called in WCM63_LG_3

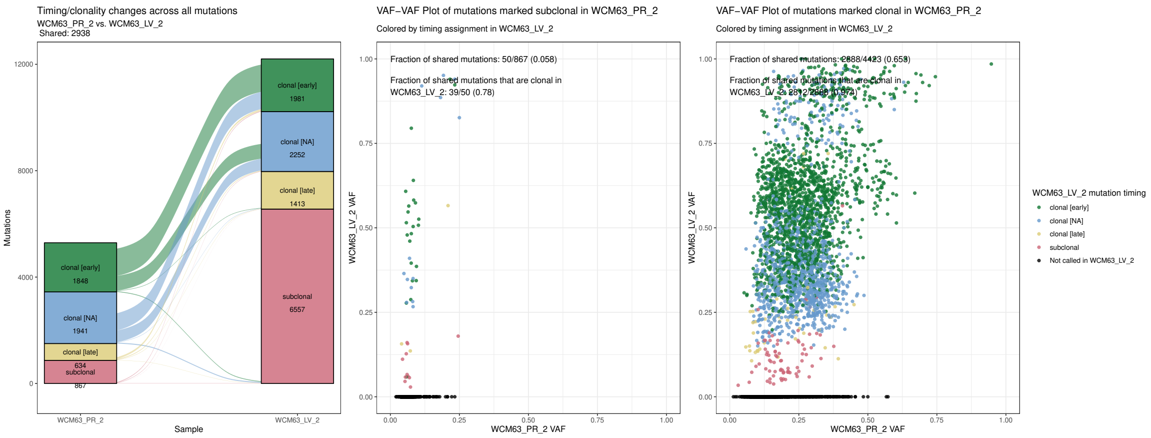
Timing/clonality changes across all mutations
WCM63_PR_2 vs. WCM63_LV_2
Shared: 2938
Mutations
Sample
WCM63_PR_2
WCM63_LV_2
clonal [early] 1848
clonal [NA] 1941
clonal [late] 634
subclonal 867
clonal [early] 1981
clonal [NA] 2252
clonal [late] 1413
subclonal 6557
VAF−VAF Plot of mutations marked subclonal in WCM63_PR_2
Colored by timing assignment in WCM63_LV_2
Fraction of shared mutations: 50/867 (0.058)
Fraction of shared mutations that are clonal in WCM63_LV_2: 39/50 (0.78)
WCM63_LV_2 VAF
WCM63_PR_2 VAF
VAF−VAF Plot of mutations marked clonal in WCM63_PR_2
Colored by timing assignment in WCM63_LV_2
WCM63_LV_2 mutation timing
clonal [early]
clonal [NA]
clonal [late]
subclonal
Not called in WCM63_LV_2

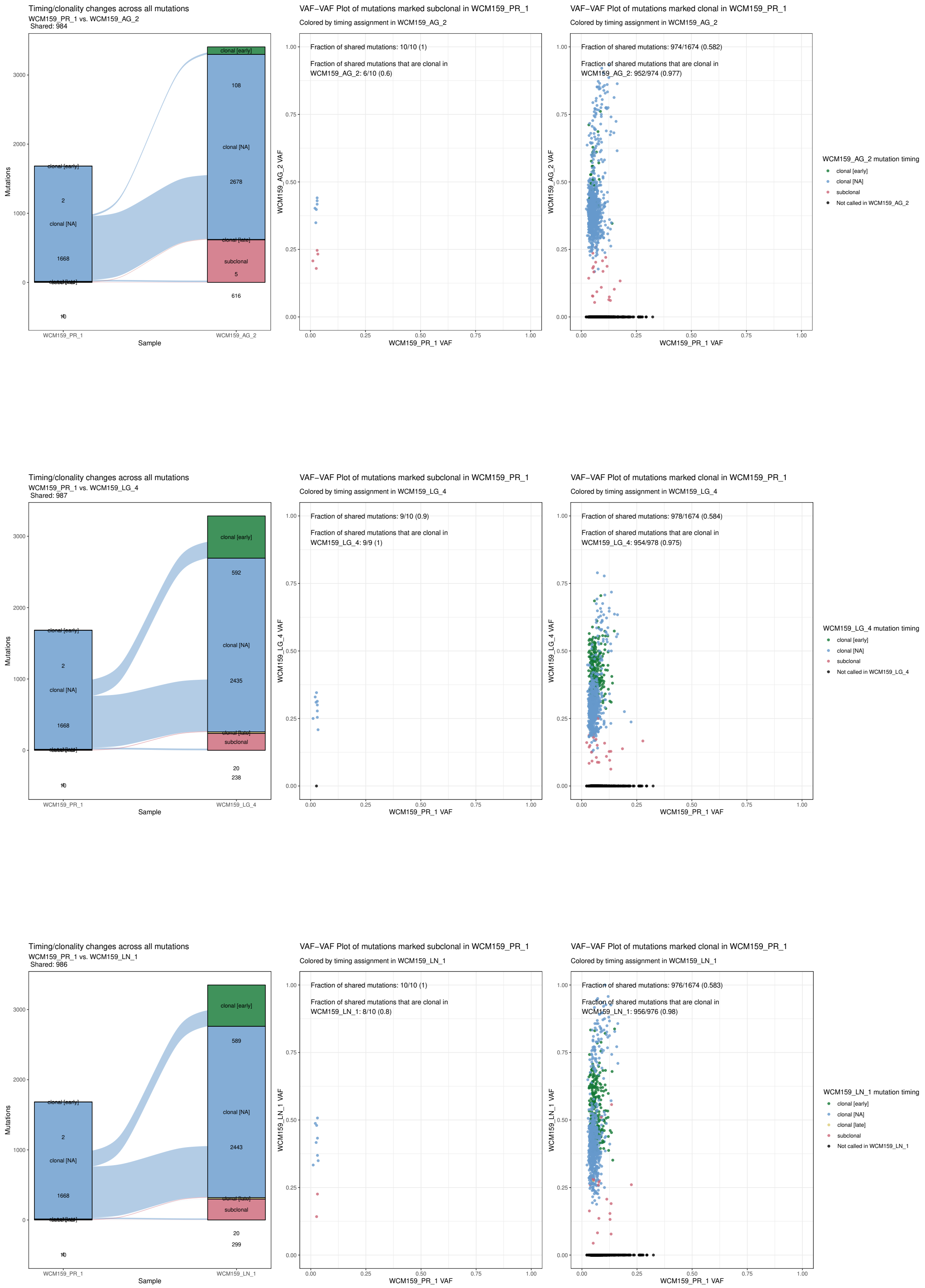
Timing/clonality changes across all mutations
WCM159_PR_1 vs. WCM159_AG_2
Shared: 984
VAF−VAF Plot of mutations marked subclonal in WCM159_PR_1
Colored by timing assignment in WCM159_AG_2
Fraction of shared mutations: 10/10 (1)
Fraction of shared mutations that are clonal in WCM159_AG_2: 6/10 (0.6)
VAF−VAF Plot of mutations marked clonal in WCM159_PR_1
Colored by timing assignment in WCM159_AG_2
Fraction of shared mutations: 974/1674 (0.582)
Fraction of shared mutations that are clonal in WCM159_AG_2: 952/974 (0.977)
WCM159_AG_2 mutation timing
clonal [early]
clonal [NA]
subclonal
Not called in WCM159_AG_2
Timing/clonality changes across all mutations
WCM159_PR_1 vs. WCM159_LG_4
Shared: 987
VAF−VAF Plot of mutations marked subclonal in WCM159_PR_1
Colored by timing assignment in WCM159_LG_4
Fraction of shared mutations: 9/10 (0.9)
Fraction of shared mutations that are clonal in WCM159_LG_4: 9/9 (1)
VAF−VAF Plot of mutations marked clonal in WCM159_PR_1
Colored by timing assignment in WCM159_LG_4
Fraction of shared mutations: 978/1674 (0.584)
Fraction of shared mutations that are clonal in WCM159_LG_4: 954/978 (0.975)
WCM159_LG_4 mutation timing
clonal [early]
clonal [NA]
subclonal
Not called in WCM159_LG_4
Timing/clonality changes across all mutations
WCM159_PR_1 vs. WCM159_LN_1
Shared: 986
VAF−VAF Plot of mutations marked subclonal in WCM159_PR_1
Colored by timing assignment in WCM159_LN_1
Fraction of shared mutations: 10/10 (1)
Fraction of shared mutations that are clonal in WCM159_LN_1: 8/10 (0.8)
VAF−VAF Plot of mutations marked clonal in WCM159_PR_1
Colored by timing assignment in WCM159_LN_1
Fraction of shared mutations: 976/1674 (0.583)
Fraction of shared mutations that are clonal in WCM159_LN_1: 956/976 (0.98)
WCM159_LN_1 mutation timing
clonal [early]
clonal [NA]
clonal [late]
subclonal
Not called in WCM159_LN_1

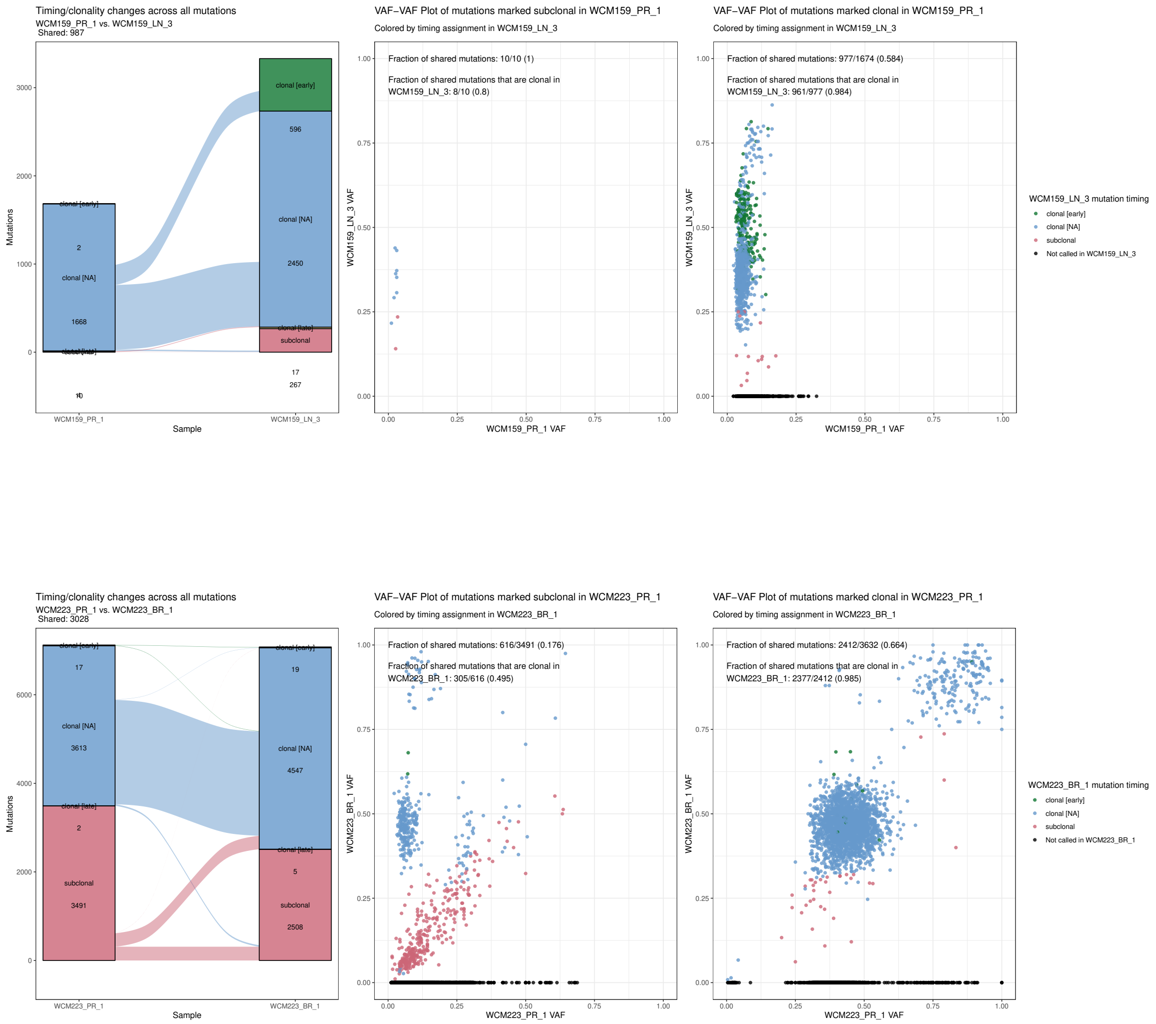

Supplementary Figure 6: Clonal relationships between primary and metastatic samples. Alluvial plot on the left indicates the clonal classification of mutations in the prostate sample (left) and the corresponding metastasis sample (right). Scatterplots display the Variant Allele Fractions of subclonal (middle plot) and clonal (right plot) mutations, colored according to their classification in the metastasis sample.

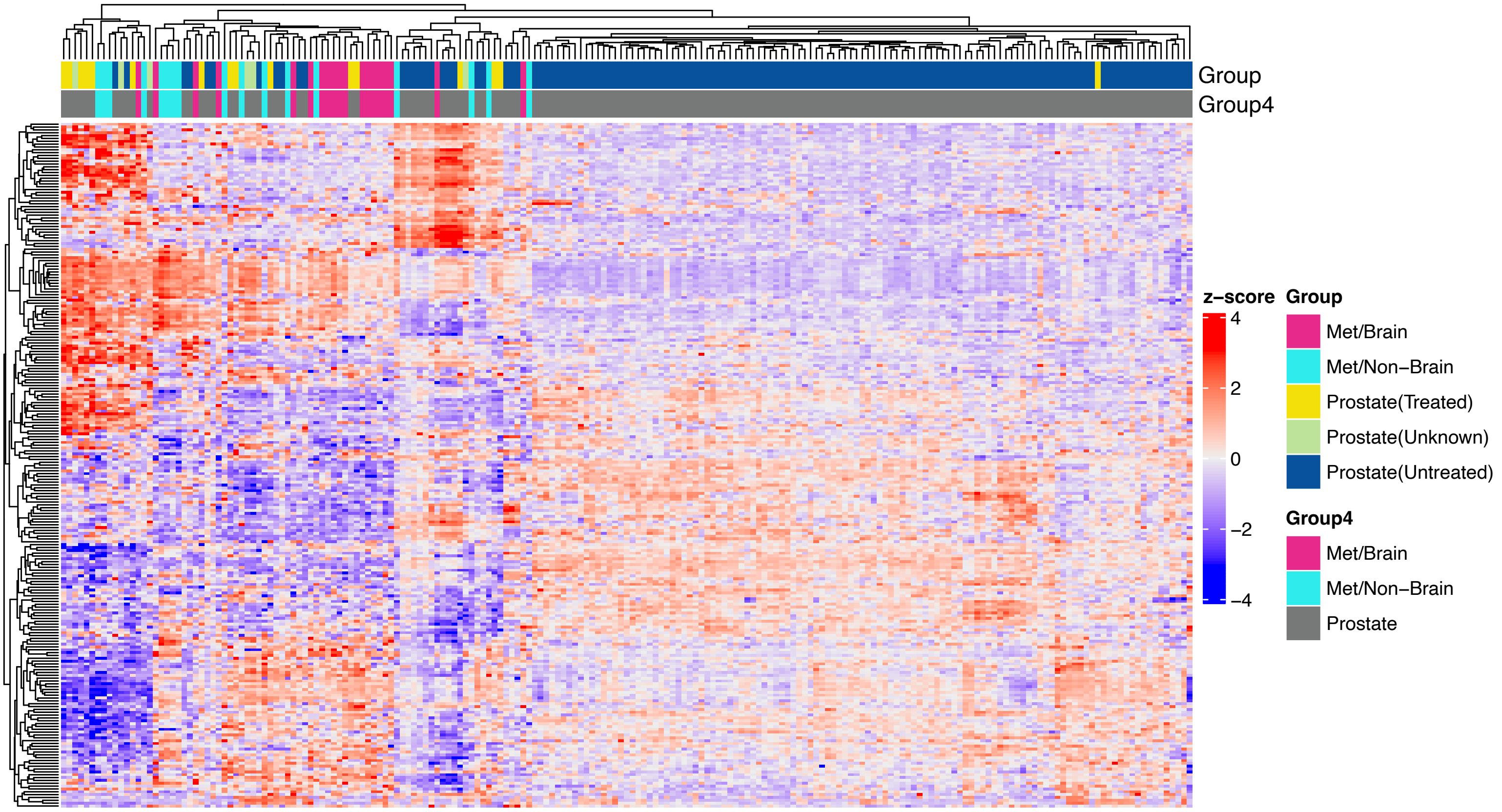

Supplementary Figure 7: Heatmap of gene expression in primary tumors, CRPC and metastatic samples.

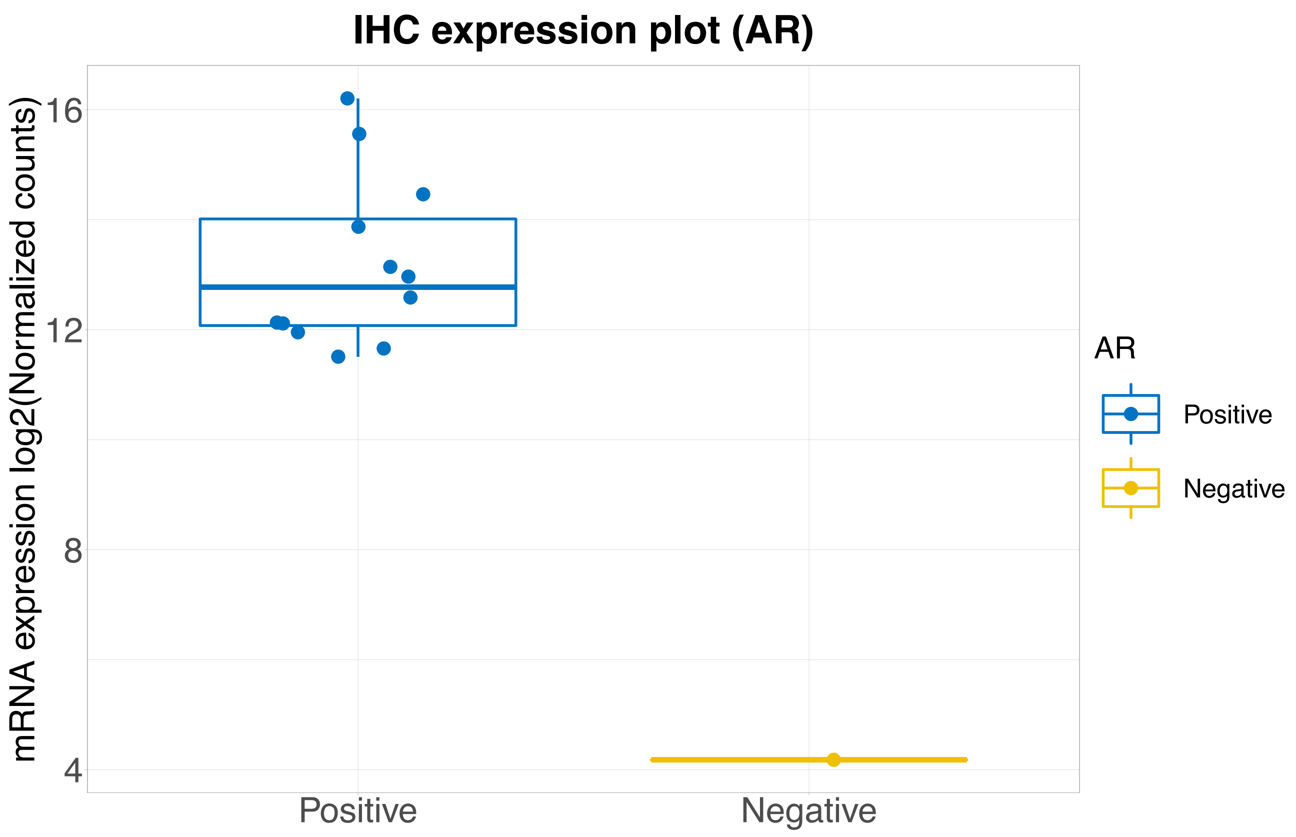
IHC expression plot (AR)
mRNA expression log2(Normalized counts)
16
12
8
4
Positive
Negative
AR
Positive
Negative

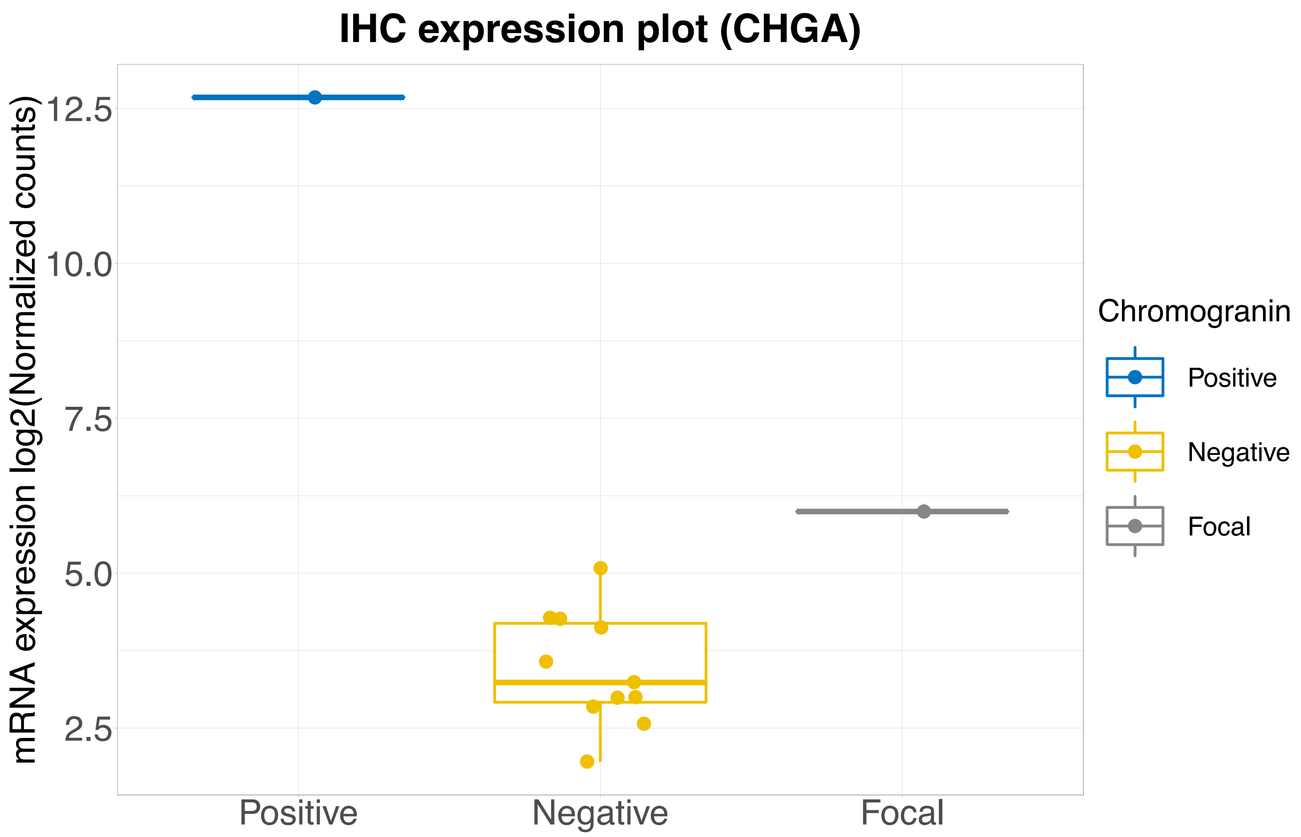
IHC expression plot (CHGA)
mRNA expression log2(Normalized counts)
12.5
10.0
7.5
5.0
2.5
Positive
Negative
Focal
Chromogranin
Positive
Negative
Focal

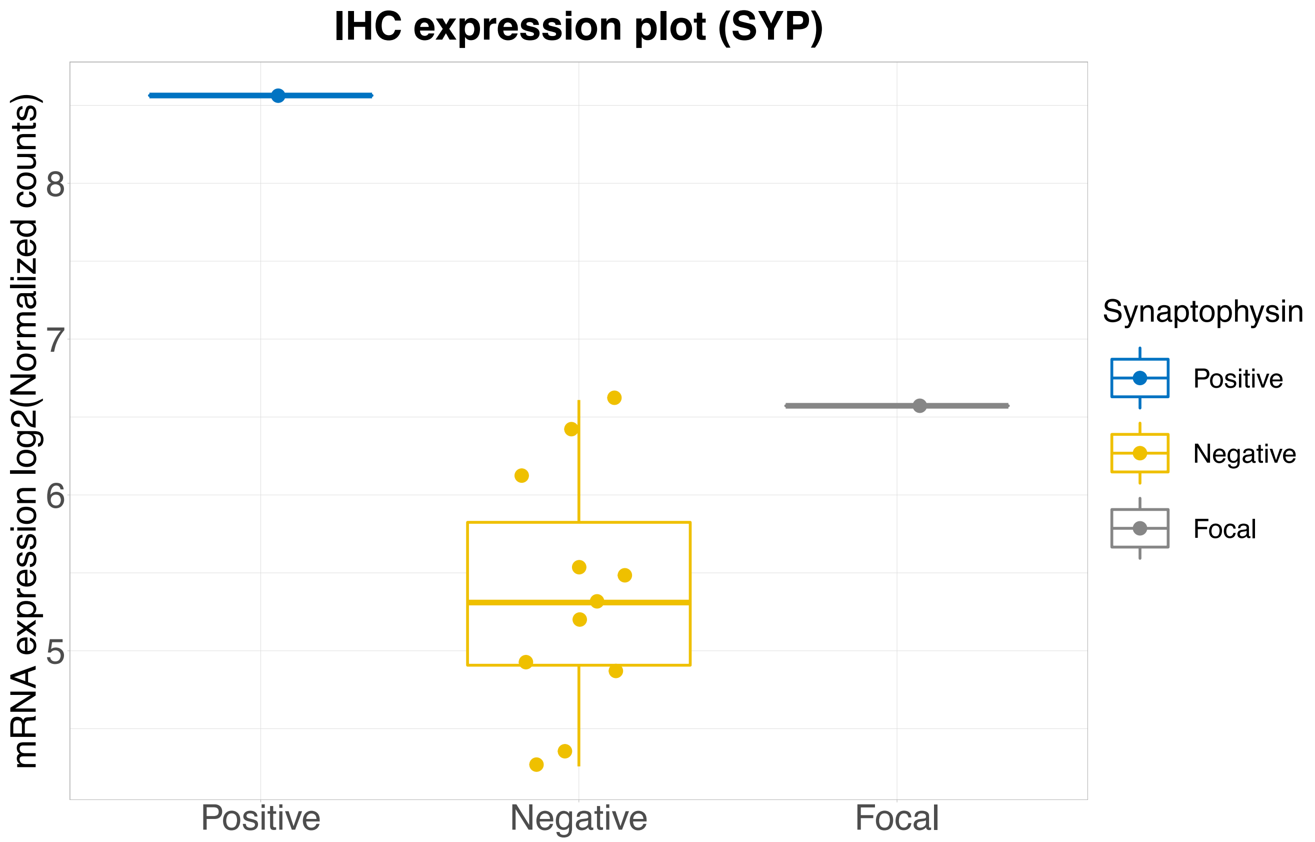
IHC expression plot (SYP)
mRNA expression log2(Normalized counts)
8
7
6
5
Positive
Negative
Focal
Synaptophysin
Positive
Negative
Focal

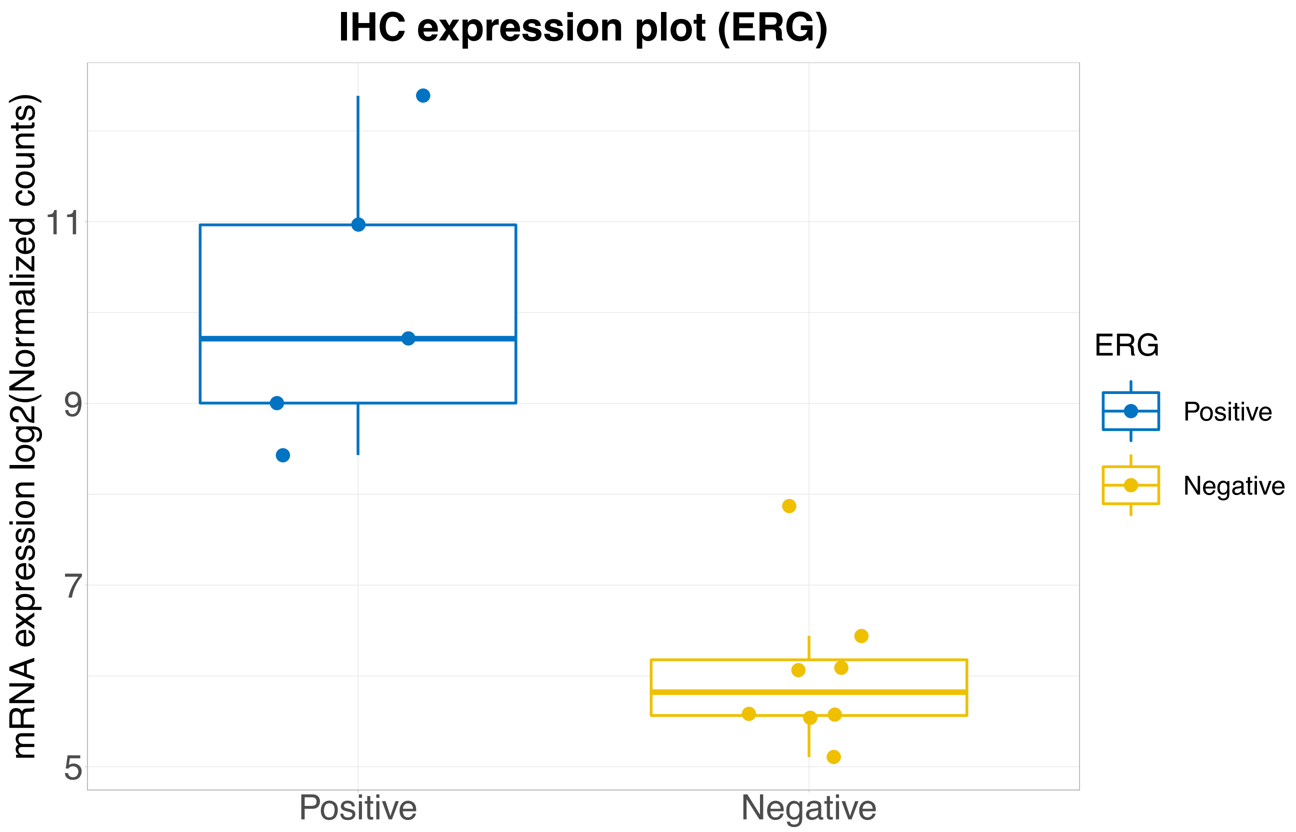
IHC expression plot (ERG)
mRNA expression log2(Normalized counts)
11
9
7
5
Positive
Negative
ERG
Positive
Negative

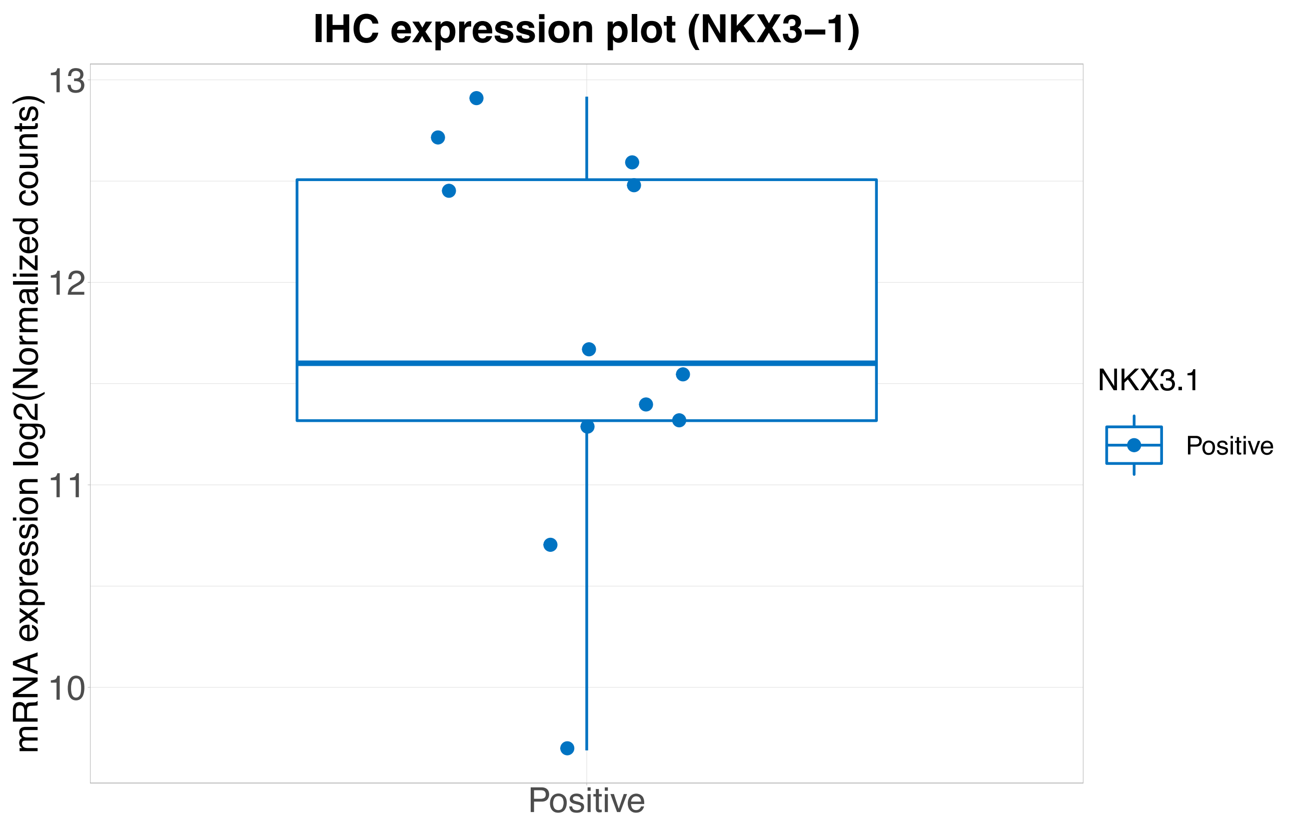

IHC expression plot (NKX3−1)
mRNA expression log2(Normalized counts)
13
12
11
10
Positive
NKX3.1
Positive

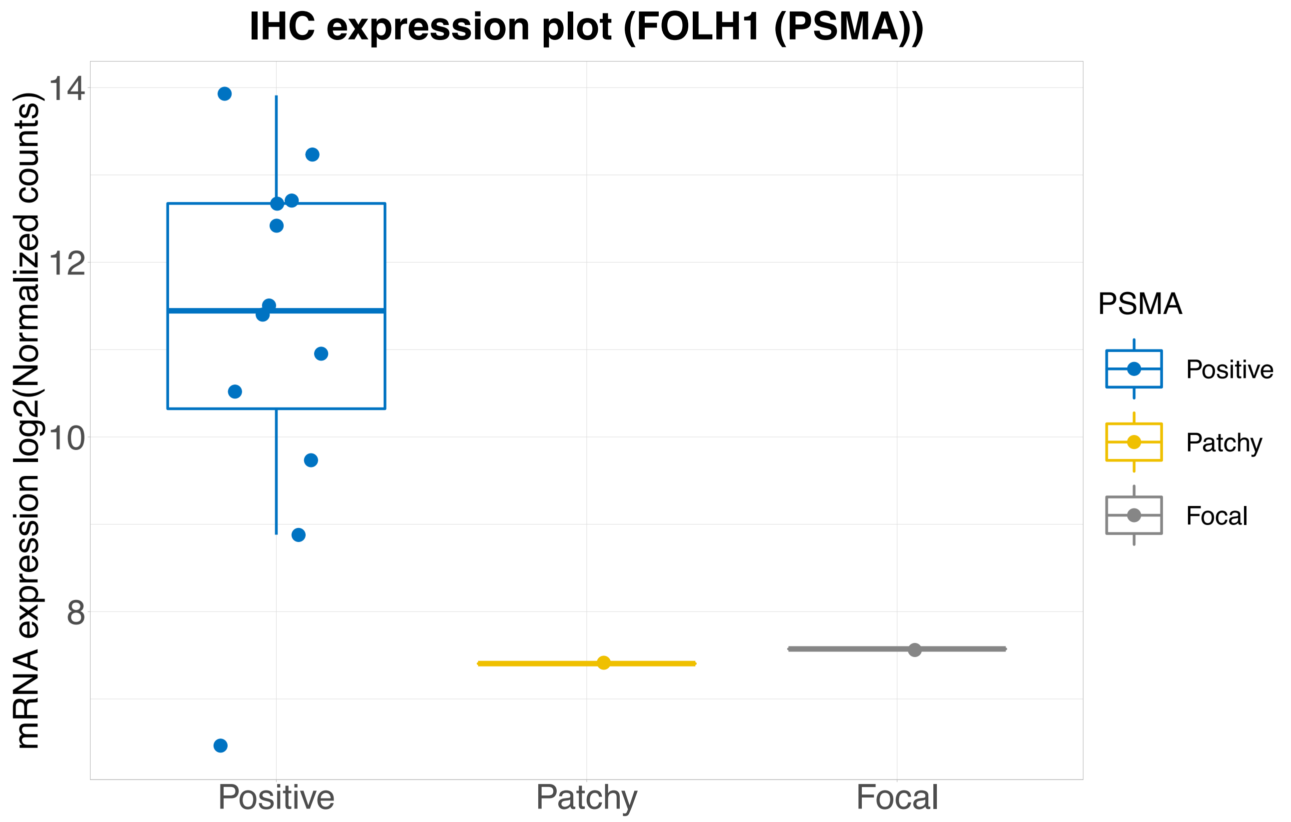

IHC expression plot (FOLH1 (PSMA))
mRNA expression log2(Normalized counts)
14
12
10
8
Positive
Patchy
Focal
PSMA
Positive
Patchy
Focal

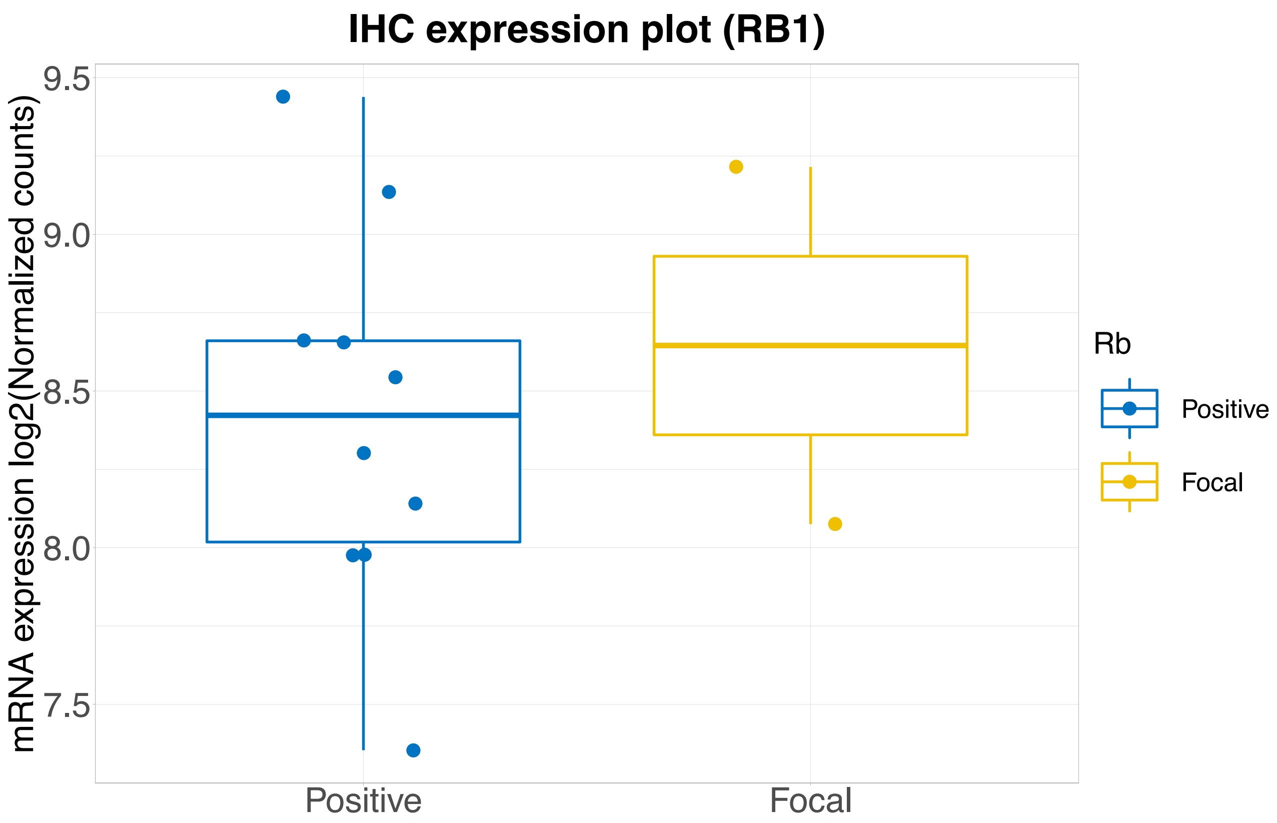

Supplementary Figure 8: Concordance of mRNA expression by Nanostring with RNAseq and expression by IHC for AR, CHGA, SYP, ERG, NKX31, PSMA, and RB protein. Each point represents a sample. The horizontal line in each box represents the median for each group. The bottom and top of each box represent the first and third quartiles, respectively. The vertical lines extend to the values no farther than 1.5 times the interquartile range.
