# Supplementary material for: Evolution of structural rearrangements in prostate cancer intracranial metastases
Source: NPJ Precis Oncol. 2023 Sep 13;7:91. doi: 10.1038/s41698-023-00435-3 (PMC10499931; doi:10.1038/s41698-023-00435-3)
Supplement: Supplementary file 4 — Reporting Summary [file 41698_2023_435_MOESM4_ESM.pdf]

nature portfolio

Corresponding author(s): Himisha Beltran
Last updated by author(s): 07/25/2023

Reporting Summary

Nature Portfolio wishes to improve the reproducibility of the work that we publish. This form provides structure for consistency and transparency in reporting. For further information on Nature Portfolio policies, see our Editorial Policies and the Editorial Policy Checklist.

Statistics

For all statistical analyses, confirm that the following items are present in the figure legend, table legend, main text, or Methods section.

n/a	Confirmed	
☐	☒	The exact sample size (*n*) for each experimental group/condition, given as a discrete number and unit of measurement
☐	☒	A statement on whether measurements were taken from distinct samples or whether the same sample was measured repeatedly
☐	☒	The statistical test(s) used AND whether they are one- or two-sided<br>*Only common tests should be described solely by name; describe more complex techniques in the Methods section.*
☐	☒	A description of all covariates tested
☐	☒	A description of any assumptions or corrections, such as tests of normality and adjustment for multiple comparisons
☐	☒	A full description of the statistical parameters including central tendency (e.g. means) or other basic estimates (e.g. regression coefficient) AND variation (e.g. standard deviation) or associated estimates of uncertainty (e.g. confidence intervals)
☐	☒	For null hypothesis testing, the test statistic (e.g. *F*, *t*, *r*) with confidence intervals, effect sizes, degrees of freedom and *P* value noted<br>*Give P values as exact values whenever suitable.*
☐	☒	For Bayesian analysis, information on the choice of priors and Markov chain Monte Carlo settings
☐	☒	For hierarchical and complex designs, identification of the appropriate level for tests and full reporting of outcomes
☐	☒	Estimates of effect sizes (e.g. Cohen's *d*, Pearson's *r*), indicating how they were calculated

Our web collection on statistics for biologists contains articles on many of the points above.

Software and code

Policy information about availability of computer code

Data collection

The open source code used in this paper were: JaBbA (https://github.com/mskilab/JaBbA), gGnome (https://github.com/mskilab/gGnome), MutationTimeR (https://github.com/gerstung-lab/MutationTimeR), deconstructSigs (https://github.com/raerose01/deconstructSigs), ShortAlignmentMarking (https://github.com/nygenome/nygc-short-alignment-marking), BWA MEM (https://github.com/lh3/bwa), GATK (https://github.com/broadinstitute/gatk), MuTect2 (https://github.com/broadinstitute/gatk), Strelka2 (https://github.com/Illumina/strelka), Lancet (https://github.com/nygenome/lancet), Svaba (https://github.com/walaj/svaba), Manta (https://github.com/Illumina/manta), Lumpy (https://github.com/arq5x/lumpy-sv), SplazerS (https://github.com/seqan/seqan/tree/master/apps/splazers), Ensembl (https://www.ensembl.org), COSMIC (https://cancer.sanger.ac.uk), COSMIC Cancer Gene Consensus (https://cancer.sanger.ac.uk/census), ClinVar (https://www.ncbi.nlm.nih.gov/clinvar/), PolyPhen (http://genetics.bwh.harvard.edu/pph2/index.shtml), SIFT (http://sift-dna.org/sift4g), FATHMM (http://fathmm.biocompute.org.uk), gnomAD (https://gnomad.broadinstitute.org/), gnomAD-SV (https://gnomad.broadinstitute.org/, https://github.com/talkowski-lab/gnomad-sv-pipeline), dbSNP (https://www.ncbi.nlm.nih.gov/snp/), Variant Effect Predictor (VEP) (http://www.ensembl.org/vep), Database of Genomic Variants (DGV) (http://dgv.tcag.ca/), AscatNGS (https://github.com/cancerit/ascatNgs), Sequenza (http://www.cbs.dtu.dk/biotools/sequenza), LICHeE (https://github.com/viq854/lichee), fragCounter (https://github.com/mskilab/fragCounter), dryclean (https://github.com/mskilab/dryclean).

Custom analysis scripts and scripts to reproduce figures are available at: https://github.com/nygenome/ProstateBrainMet_WGS_paper_figures
The open source code used in this paper were: JaBbA (https://github.com/mskilab/JaBbA), gGnome (https://github.com/mskilab/gGnome), MutationTimeR (https://github.com/gerstung-lab/MutationTimeR), deconstructSigs (https://github.com/raerose01/deconstructSigs), ShortAlignmentMarking (https://github.com/nygenome/nygc-short-alignment-marking), BWA MEM (https://github.com/lh3/bwa), GATK (https://github.com/broadinstitute/gatk), MuTect2 (https://github.com/broadinstitute/gatk), Strelka2 (https://github.com/Illumina/strelka), Lancet (https://github.com/nygenome/lancet), Svaba (https://github.com/walaj/svaba), Manta (https://github.com/Illumina/manta), Lumpy

(https://github.com/arq5x/lumpy-sv), SplazerS (https://github.com/seqan/seqan/tree/master/apps/splazers), Ensembl (https://www.ensembl.org), COSMIC (https://cancer.sanger.ac.uk), COSMIC Cancer Gene Consensus (https://cancer.sanger.ac.uk/census), ClinVar (https://www.ncbi.nlm.nih.gov/clinvar/), PolyPhen (http://genetics.bwh.harvard.edu/pph2/index.shtml), SIFT (http://sift-dna.org/sift4g), FATHMM (http://fathmm.biocompute.org.uk), gnomAD (https://gnomad.broadinstitute.org/), gnomAD-SV (https://gnomad.broadinstitute.org/, https://github.com/talkowski-lab/gnomad-sv-pipeline), dbSNP (https://www.ncbi.nlm.nih.gov/snp/), Variant Effect Predictor (VEP) (http://www.ensembl.org/vep), Database of Genomic Variants (DGV) (http://dgv.tcag.ca/), AscatNGS (https://github.com/cancerit/ascatNgs), Sequenza (http://www.cbs.dtu.dk/biotools/sequenza), LICHeE (https://github.com/viq854/lichee), fragCounter (https://github.com/mskilab/fragCounter), dryclean (https://github.com/mskilab/dryclean).

Data analysis: Custom analysis scripts and scripts to reproduce figures are available at: https://github.com/nygenome/ProstateBrainMet_WGS_paper_figures

For manuscripts utilizing custom algorithms or software that are central to the research but not yet described in published literature, software must be made available to editors and reviewers. We strongly encourage code deposition in a community repository (e.g. GitHub). See the Nature Portfolio guidelines for submitting code & software for further information.

Data

Policy information about availability of data

All manuscripts must include a data availability statement. This statement should provide the following information, where applicable:

- Accession codes, unique identifiers, or web links for publicly available datasets
- A description of any restrictions on data availability
- For clinical datasets or third party data, please ensure that the statement adheres to our policy

Data is accessible via dbGaP (accession #XXX)

Research involving human participants, their data, or biological material

Policy information about studies with human participants or human data. See also policy information about sex, gender (identity/presentation), and sexual orientation and race, ethnicity and racism.

Reporting on sex and gender	36 male patients with prostate cancer and intracranial metastases
Reporting on race, ethnicity, or other socially relevant groupings	Race and ethnicity not available
Population characteristics	Patient with prostate cancer and intracranial metastases were retrospectively identified
Recruitment	Patients with intracranial metastases were retrospectively identified through institutional databases and tumors were collected retrospectively or prospectively at the time of surgery/autopsy with written informed consent.
Ethics oversight	Tumor and blood specimens were evaluated through protocols approved by the Weill Cornell Medicine (IRB #1610017620), Dana-Farber Cancer Institute (IRB #19-883), University of North Carolina (UNC IRB #08-0242) and Oregon Health Sciences (IRB #00019876) Institutional Review Boards (IRB #19-883, #1305013903).

Note that full information on the approval of the study protocol must also be provided in the manuscript.

Field-specific reporting

Please select the one below that is the best fit for your research. If you are not sure, read the appropriate sections before making your selection.

☒ Life sciences ☐ Behavioural & social sciences ☐ Ecological, evolutionary & environmental sciences

For a reference copy of the document with all sections, see nature.com/documents/nr-reporting-summary-flat.pdf

Life sciences study design

All studies must disclose on these points even when the disclosure is negative.

Sample size	36 patients
Data exclusions	20 pts had metastatic intracranial tumor tissue available for molecular studies
Replication	N/A
Randomization	N/A
Blinding	N/A

Reporting for specific materials, systems and methods

We require information from authors about some types of materials, experimental systems and methods used in many studies. Here, indicate whether each material, system or method listed is relevant to your study. If you are not sure if a list item applies to your research, read the appropriate section before selecting a response.

Materials & experimental systems

n/a	Involved in the study
☐	☐ Antibodies
☐	☐ Eukaryotic cell lines
☐	☐ Palaeontology and archaeology
☐	☐ Animals and other organisms
☐	☒ Clinical data
☐	☐ Dual use research of concern
☐	☐ Plants

Methods

n/a	Involved in the study
☐	☐ ChIP-seq
☐	☐ Flow cytometry
☐	☐ MRI-based neuroimaging

Antibodies

Antibodies used	NKX3.1 (clone Rabbit Polyclonal, Biocare Medical), AR (clone F39.4.1, Biogenex), PSMA (clone 3E6, Dako), chromogranin (clone FH7, Leica), synaptophysin (clone 27G12, Leica), and ERG (EPR3864, Abcam)
Validation	N/A

Eukaryotic cell lines

Policy information about cell lines and Sex and Gender in Research

Cell line source(s)	N/A
Authentication	N/A
Mycoplasma contamination	N/A
Commonly misidentified lines (See ICLAC register)	N/A

Palaeontology and Archaeology

Specimen provenance	N/A
Specimen deposition	N/A
Dating methods	N/A

☐ Tick this box to confirm that the raw and calibrated dates are available in the paper or in Supplementary Information.

Ethics oversight	N/A

Note that full information on the approval of the study protocol must also be provided in the manuscript.

Animals and other research organisms

Policy information about studies involving animals; ARRIVE guidelines recommended for reporting animal research, and Sex and Gender in Research

Laboratory animals	N/A
Wild animals	N/A
Reporting on sex	N/A
Field-collected samples	*For laboratory work with field-collected samples, describe all relevant parameters such as housing, maintenance, temperature, photoperiod and end-of-experiment protocol OR state that the study did not involve samples collected from the field.*

Ethics oversight	N/A

Note that full information on the approval of the study protocol must also be provided in the manuscript.

Clinical data

Policy information about clinical studies

All manuscripts should comply with the ICMJE guidelines for publication of clinical research and a completed CONSORT checklist must be included with all submissions.

Clinical trial registration	N/A
Study protocol	N/A
Data collection	2003-2018
Outcomes	overall survival

Dual use research of concern

Policy information about dual use research of concern

Hazards

Could the accidental, deliberate or reckless misuse of agents or technologies generated in the work, or the application of information presented in the manuscript, pose a threat to:

No	Yes	
☒	☐	Public health
☒	☐	National security
☒	☐	Crops and/or livestock
☒	☐	Ecosystems
☒	☐	Any other significant area

Experiments of concern

Does the work involve any of these experiments of concern:

No	Yes	
☒	☐	Demonstrate how to render a vaccine ineffective
☒	☐	Confer resistance to therapeutically useful antibiotics or antiviral agents
☒	☐	Enhance the virulence of a pathogen or render a nonpathogen virulent
☒	☐	Increase transmissibility of a pathogen
☒	☐	Alter the host range of a pathogen
☒	☐	Enable evasion of diagnostic/detection modalities
☒	☐	Enable the weaponization of a biological agent or toxin
☒	☐	Any other potentially harmful combination of experiments and agents

Plants

Seed stocks	N/A
Novel plant genotypes	N/A
Authentication	N/A

ChIP-seq

Data deposition

☐ Confirm that both raw and final processed data have been deposited in a public database such as GEO.

☐ Confirm that you have deposited or provided access to graph files (e.g. BED files) for the called peaks.

Data access links<br>*May remain private before publication.*	N/A
Files in database submission	N/A
Genome browser session<br>(e.g. UCSC)	N/A

Methodology

Replicates	N/A
Sequencing depth	N/A
Antibodies	N/A
Peak calling parameters	N/A
Data quality	N/A
Software	N/A

Flow Cytometry

Plots

Confirm that:

- [ ] The axis labels state the marker and fluorochrome used (e.g. CD4-FITC).
- [ ] The axis scales are clearly visible. Include numbers along axes only for bottom left plot of group (a 'group' is an analysis of identical markers).
- [ ] All plots are contour plots with outliers or pseudocolor plots.
- [ ] A numerical value for number of cells or percentage (with statistics) is provided.

Methodology

Sample preparation	N/A
Instrument	N/A
Software	N/A
Cell population abundance	N/A
Gating strategy	N/A

- [ ] Tick this box to confirm that a figure exemplifying the gating strategy is provided in the Supplementary Information.

Magnetic resonance imaging

Experimental design

Design type	N/A
Design specifications	N/A
Behavioral performance measures	N/A

Acquisition

Imaging type(s)	N/A
Field strength	N/A
Sequence & imaging parameters	N/A
Area of acquisition	N/A

Diffusion MRI ☐ Used ☐ Not used

Preprocessing

Preprocessing software	N/A
Normalization	N/A
Normalization template	N/A
Noise and artifact removal	N/A
Volume censoring	N/A

Statistical modeling & inference

Model type and settings	N/A
Effect(s) tested	N/A

Specify type of analysis: ☐ Whole brain ☐ ROI-based ☐ Both

Statistic type for inference (See Eklund et al. 2016)	N/A
Correction	N/A

Models & analysis

n/a	Involved in the study
☐	☐ Functional and/or effective connectivity
☐	☐ Graph analysis
☐	☐ Multivariate modeling or predictive analysis

Functional and/or effective connectivity	N/A
Graph analysis	N/A
Multivariate modeling and predictive analysis	N/A
